# Phylogenetic Approach Reveals That Virus Genotype Largely Determines HIV Set-Point Viral Load

**DOI:** 10.1371/journal.ppat.1001123

**Published:** 2010-09-30

**Authors:** Samuel Alizon, Viktor von Wyl, Tanja Stadler, Roger D. Kouyos, Sabine Yerly, Bernard Hirschel, Jürg Böni, Cyril Shah, Thomas Klimkait, Hansjakob Furrer, Andri Rauch, Pietro L. Vernazza, Enos Bernasconi, Manuel Battegay, Philippe Bürgisser, Amalio Telenti, Huldrych F. Günthard, Sebastian Bonhoeffer, the Swiss HIV Cohort Study

**Affiliations:** 1 Institute of Integrative Biology, ETH Zürich, Zürich, Switzerland; 2 Division of Infectious Diseases and Hospital Epidemiology, University Hospital of Zürich, University of Zürich, Zürich, Switzerland; 3 Central Laboratory of Virology, University Hospital of Geneva, Geneva, Switzerland; 4 Division of Infectious Diseases, University Hospital of Geneva, Geneva, Switzerland; 5 Swiss National Center for Retroviruses, Institute of Medical Virology, University of Zürich, Zürich, Switzerland; 6 Institute of Medical Microbiology, University of Basel, Basel, Switzerland; 7 University Clinic of Infectious Diseases, University Hospital of Bern, University of Bern, Bern, Switzerland; 8 Division of Infectious Diseases, Cantonal Hospital of St. Gallen, St. Gallen, Switzerland; 9 Division of Infectious Diseases, Regional Hospital of Lugano, Lugano, Switzerland; 10 Division of Infectious Diseases and Hospital Epidemiology, University Hospital of Basel, Basel, Switzerland; 11 Division of Immunology and Allergy, University Hospital of Lausanne, Lausanne, Switzerland; 12 Institute of Microbiology, University Hospital of Lausanne, Lausanne, Switzerland; University of Texas at Austin, United States of America

## Abstract

HIV virulence, i.e. the time of progression to AIDS, varies greatly among patients. As for other rapidly evolving pathogens of humans, it is difficult to know if this variance is controlled by the genotype of the host or that of the virus because the transmission chain is usually unknown. We apply the phylogenetic comparative approach (PCA) to estimate the heritability of a trait from one infection to the next, which indicates the control of the virus genotype over this trait. The idea is to use viral RNA sequences obtained from patients infected by HIV-1 subtype B to build a phylogeny, which approximately reflects the transmission chain. Heritability is measured statistically as the propensity for patients close in the phylogeny to exhibit similar infection trait values. The approach reveals that up to half of the variance in set-point viral load, a trait associated with virulence, can be heritable. Our estimate is significant and robust to noise in the phylogeny. We also check for the consistency of our approach by showing that a trait related to drug resistance is almost entirely heritable. Finally, we show the importance of taking into account the transmission chain when estimating correlations between infection traits. The fact that HIV virulence is, at least partially, heritable from one infection to the next has clinical and epidemiological implications. The difference between earlier studies and ours comes from the quality of our dataset and from the power of the PCA, which can be applied to large datasets and accounts for within-host evolution. The PCA opens new perspectives for approaches linking clinical data and evolutionary biology because it can be extended to study other traits or other infectious diseases.

## Author Summary

Some untreated patients infected by HIV die within a couple of years, while others survive more than 25 years. To date, it is still unclear whether this variance in the virulence of the infection is due to the host or to the virus genotype. One of the main difficulties in answering this question is that, as for most human diseases, we tend not to know who infected whom. Here, we solve this problem by adopting a phylogenetic approach, which estimates the heritability of species traits on a phylogeny. In our case, species correspond to infected patients and the trait is an infection trait. The phylogeny is obtained from the HIV RNA sequences isolated in each patient. We find that more than half of the variance observed in the set-point viral load—a trait that predicts virulence—is heritable from one infection to the next. This implies that set-point viral load is strongly controlled by the virus genotype. This application of the phylogenetic comparative approach to infectious diseases yields major results for the deciphering of HIV pathogenesis. Future applications to other traits and/or other pathogens will help us to better understand rapidly evolving diseases of humans.

## Introduction

One of the hallmarks of HIV infection is the enormous variation in disease progression. While some untreated patients survive for more than 25 years, others die within a year of infection [1]. Delineating what fraction of this variation is attributable to host versus virus genotype has important clinical and epidemiological applications. Whole genome association studies showed that at least 15% of the variations in traits associated with disease progression in HIV can be explained by common host genetic variants [2], [3]. In this study, we adopt a new approach to quantify the contribution of the viral genotype to disease progression.

The natural course of an HIV infection is divided into three clinical stages: the acute phase, which lasts for several weeks; the asymptomatic phase, which can last several years; and the AIDS phase, which can last several months to a few years and ends with the death of the patient. The effect of the HIV viral genotype on disease progression is debated. Arguably, this is due to the fact that, as for most infectious diseases of humans, it is difficult to determine the transmission chain (i.e. ‘Who infected whom?’). Early studies showed that long times to AIDS are associated with the receipt of blood from donors who developed AIDS late [4], [5]. A study found a strong correlation between maternal and infant viral load [6], but mother-to-child transmission introduces confounding factors through host genetic relatedness. In some cases, transmission pairs are known and one can then measure the heritability of traits from one infection to the next. This heritability corresponds to the fraction of the variance among patients that is explained by the virus genotype [7], [8]. Studies found that 21% [9], 23% [8], 25% [10] and 55% [11] of the variance in set-point viral load (spVL) can be explained by the virus genotype. These studies suggest that infection traits can be heritable but the accuracy of their estimates is likely to be limited by the sample size of the data and the accuracy of their spVL measurement.

The phylogenetic comparative approach (PCA) allows to estimate the phylogenetic signal for a trait measured in several species if the species' phylogeny is known. This signal indicates the extent to which the phylogeny explains observed trait values (see the Methods). Several studies show that this signal is very similar to heritability [12], [13], which we also verify in this study. Here, we use this classical evolutionary biology method [14] to address epidemiological questions.

We consider the case of HIV infection traits using the data from the Swiss HIV Cohort Study (SHCS [15]). As the majority of previous studies [2], [3], [6], [8], [9], [16], [17], we focus on the set-point viral load (spVL, see the Methods) measured during the asymptomatic phase of an HIV infection because it has been shown to be associated with the time to AIDS, i.e. virulence [16], [18]–[20]. The quality of the SHCS patient data allows us to have a better estimation of spVL than many studies that often rely on single viral load measurements to estimate spVL (see the Materials and Methods). Further, we consider the decline slope of the CD4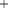 T-cells (dsCD4), which also predicts virulence [21], but to a smaller extent than spVL [20].

**Figure 1:**
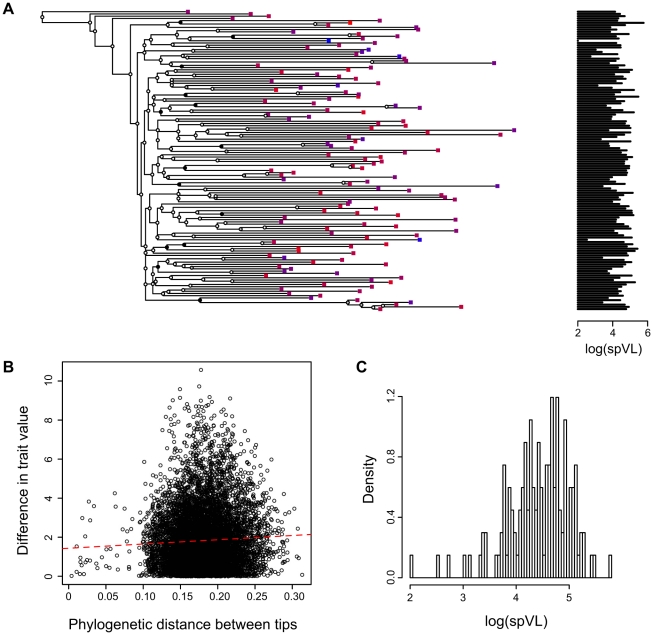
Combining phylogenies and trait values in the MSM strict dataset. **A**) A phylogeny based on HIV sequences obtained from patients with known set-point viral loads, **B**) Phylogenetic distance between two tips versus difference in trait value between these two tips (slope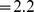 and p-value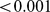) and **C**) Distribution of log(spVL) values in the MSM strict dataset. Panel A shows the maximum likelihood phylogenetic tree built with the MSM strict dataset. Squares on the tips of the tree correspond to infected patients. The colour of the squares and the graph on the right indicate the set-point viral load (colours range from blue to red for increasing log(spVL)). The PCA tests the correlation between proximity in the phylogeny and trait values (log(spVL)). The circles on the tree nodes indicate bootstrap values: black is greater than 90%, grey is between 50 and 90% and white is lower than 50%.

We use sequences of the HIV *pol* gene isolated from infected patients to build a phylogenetic tree, where each leaf corresponds to a patient whose trait values are known (Figure 1). Proximity in the resulting tree reflects proximity in the transmission chain [22], [23] but we emphasise that the phylogeny is only an approximation of the true transmission history. As we will show later on, our approach has the advantage of being robust to this noise in the phylogeny. We then quantify phylogenetic signal for each of the traits on the tree [24]. In addition to log(spVL) and dsCD4, we study a third trait as a control: the probability for resistance to zidovudine (AZT), denoted prAZT. We expect the latter trait to be strongly heritable because it is evaluated from the virus *pol* sequence. Note that there is no overlap between the information used to build the phylogeny and that used to evaluate prAZT (see the Methods).

We split the data into 4 datasets using 2 criteria. First, in addition to the ‘strict’ definition used in previous studies to define spVL from multiple viral load measurements [2], [3], we also consider a ‘liberal’ definition (see the Methods). The strict definition is known to improve the accuracy of the spVL measure and we want to assess the importance of the quality of this estimate on our ability to detect heritability. Second, we study one of the transmission groups (‘men having sex with men’, or MSM) separately. This group is likely to yield a more accurate phylogeny because of denser sampling in the SHCS [25]. We show in Supplementary Results (Text S1) that focusing on these datasets also removes some of the confounding factors, such as patient sex, transmission group or age, on infection trait values.

We quantify phylogenetic signal using two estimators (denoted 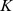 [12] and 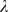 [26]) that are based on two different methods to better detect potential artifactual values (see the Methods). A signal value of 0 implies that the phylogeny does not contain any information to explain the variance of the trait in the population. Conversely, a signal value of 1 means that the distance between tips in the phylogeny used best explains the tip data assuming a Brownian model of evolution of the trait on the tree.

## Results

### Trait heritability

We first present the results obtained with the ‘MSM strict’ dataset. This dataset is the smallest we study. It has the advantage of relying on the criterion for spVL already used by earlier studies and of minimising noise in the data linked with transmission groups. The estimator for phylogenetic signal 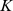 reveals a significantly high signal for log(spVL) (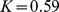, Table 1). The standard deviation estimated from the bootstrapped trees is 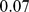.

**Table 1:** Phylogenetic signal in the 4 datasets for the three different traits.

Dataset	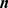	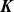 for log(spVL)	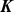 for dsCD4	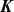 for prAZT	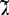 for log(spVL)	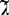 for dsCD4	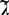 for prAZT
MSM strict	134	0.59 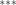	n.s.	0.91 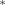	0.51 (0.27)	0	1.07 (0.12)
all strict	230	n.s.	n.s.	n.s.	0.17	0	0.88 (0.06)
MSM liberal	404	0.09 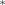	n.s.	0.82 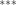	0.13 (0.05)	0	1.07 (0.015)
all liberal	661	n.s.	n.s.	0.71 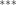	—	—	—

We use two estimators (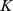 and 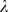) that lead to similar results. Significance code for the p-value of the randomisation test for 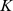 is ‘
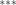
’ 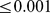, ‘
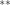
’ 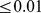, ‘
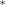
’ 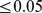, and ‘n.s.’ indicates that the signal does not differ from that found on a random tree. The 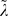 are obtained by taking the median value of 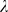 over 161 trees (see the Methods). We also show the standard deviation in brackets. ‘—’ indicates that the largest tree could not be computed with the Bayesian method because of the large number of patients. 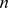 is the sample size of each dataset.

As explained above, uncertainty is inherent to any HIV phylogeny. Therefore, one might argue that the value we find is underestimated because of the noise in the phylogeny. Improving a phylogeny is a difficult task but making it worse is easy. We thus check for the robustness of the value of 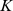 by introducing errors in the phylogeny in two ways. The first way consists in measuring 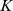 on the bootstrapped trees, which are by definition less accurate than the consensus tree. The second way consists in swapping 3, 10 or 20% of the tip values at random. As shown in the Supplementary Results, on average considering the trees from the bootstrap or trees with errors does not significantly affect the signal intensity (i.e. the value of 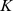): with more noise, the value of 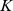 remains constant but the significance level decreases.

The intensity of the signal strongly decreases when we use the liberal criterion for spVL (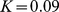 in the ‘MSM liberal’ dataset), which highlights the importance of the accuracy of the spVL estimate. The signal even becomes non-significant when we consider all the transmission groups. This can be explained by the fact that, in the SHCS, patients from the MSM transmission group tend to cluster on a phylogeny and are more densely sampled than patients from other transmission groups (see the Methods). As a consequence, a phylogeny built only on sequences from MSM patients is likely to be closer to the transmission chain. Also, using the liberal criterion or considering all the transmission groups means that log(spVL) is affected by confounding factors.

Results show no phylogenetic signal for dsCD4 in all datasets (Table 1). A possibility is that CD4 densities vary strongly within patients on short time scales. However, even a less variable estimate, the decline slope of the ratio of CD4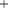 to CD8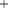 T-cells, did not exhibit significant phylogenetic signal. This suggests that this trait is not directly affected by the viral genotype or that the effect is too weak to be detected.

For prAZT in the MSM strict dataset, we find 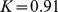 (with a standard deviation on bootstrapped trees of 0.084). As for the log(spVL), this result is robust to noise in the phylogeny (see Supplementary Results). Using the liberal criterion has little effect on this value (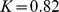 in the MSM lib dataset), which is consistent with the fact that drug resistance is genetically determined. The drop in heritability is likely to be a consequence of the increase in tree size, which decreases the accuracy of 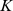 (see Supplementary Results). When we consider all the transmission groups, we find a non-significant value for the strict dataset and 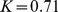 in the liberal dataset. This could be due to the fact that average prAZT is higher in one of the transmission groups (injection drug users) and to the fact that a phylogeny built with MSM patients better reflects the transmission chain.

We also perform these measures using a different estimator for phylogenetic signal, 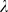 [26]. Results are similar to those found with 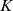 (Table 1).

Many studies argue that phylogenetic signal can be interpreted as a phylogenetic heritability [12], [13]. At first, this may seem problematic because phylogenetic signal is measured for different species, whereas heritability is defined in population genetics as the ratio between the genetic and the phenotypic variance observed for a trait in a given population [7]. Working on traits of infectious diseases bridges phylogenetics and population genetics because it involves a population of infected individuals instead of a collection of species. This would also be the case for any phylogenetic approach based on a phylogeny of individuals.

**Figure 2:**
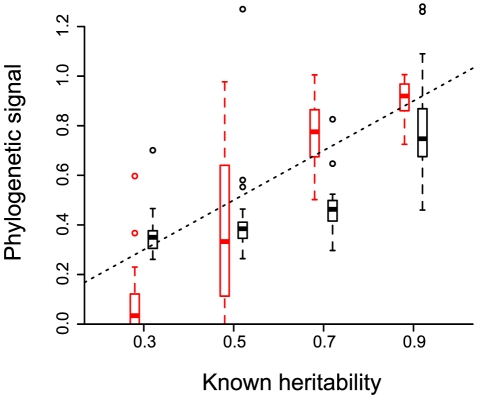
Phylogenetic signal estimated for evolutionary processes with known heritability. 20 phylogenies are simulated to model the evolution of an infection trait in a case where heritability is set to a given value. Phylogenetic signal (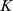 in black and 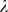 in red) is then estimated on each tree using only 128 leaves to account for incomplete sampling. The box plot shows the median values, the three quartiles and the outliers. The dashed line shows 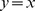. The slope is 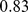 (p-value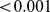 and adjusted 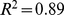) for 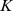 and of 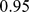 (p-value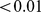 and adjusted 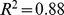) for 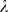.

In order to check that there indeed is an equivalency between the concepts of heritability and phylogenetic signal, we simulate phylogenetic trees reflecting the evolution of a trait under an evolutionary process with known heritability (see the Methods). The values of 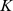 (black) and 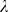 (red) estimated on these simulated trees strongly correlate with known heritability (Figure 2). Note that 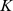 performs better than 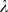 when the heritability is low. Furthermore, the variance in 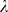 is much greater, which is why we only estimate it as a median value over many phylogenies (the posterior tree distribution). The simulations confirm that estimating phylogenetic signal for a trait yields useful quantitative estimates of trait heritability.

### Correlation among traits

The phylogenetic comparative approach (PCA) is widely used to study correlations among traits because taxa are not independent and comparisons between traits should be weighted according to the distance between taxa in the phylogeny [12]–[14], [24], [26], [27]. These ideas can be applied to infection trait values.

In Table 2, we show the difference between a classical regression between two traits and a regression that incorporates the non-independence of the data for HIV infection traits. The slope of the regression between spVL and dsCD4 decreases by approximately 60% when the phylogeny is taken into account. Furthermore, spVL does not explain much of the variance in dsCD4 (a linear model yields an adjusted 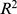 of 0.047). We also find that accounting for the non-independence of the data can alter the significance of a correlation: the dsCD4 varies among transmission groups with a classical test but not with a test including the phylogeny. This provides new data to the recent debate on the link between these two predictors of virulence (see [28] and the resulting correspondence).

**Table 2:** Regressions between life-history traits with and without correction for phylogenetic signal.

Traits and dataset	Test	Slope (SE)	Y-intercept (SE)	ln(likelihood)	AIC
log(spVL) vs. dsCD4 (*MSM strict*)	OLS	−1.7e-3 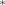 (8.1e-4)	4.26 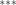 (0.08)	−56	119
	RegBM	−2.7e-3 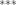 (6.8e-4)	4.1 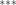 (0.34)	−57	121
Trait variation among risk groups (*all liberal*)	OLS	log(spVL) 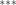 and dsCD4 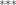			
	RegBM	log(spVL) 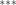 and dsCD4 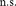			

The first line is a regression between log(spVL) and dsCD4. The second line tests if values for a given trait vary across risk groups. OLS is the ordinary generalised least square without phylogenetic correction (a generalised linear model yielded similar results); RegBM indicates a correction based on the tree assuming Brownian motion. SE stands for ‘Standard Error’. For further details, see the Supplementary Methods. Significance code for the p-value of the test is ‘
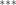
’ 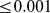, ‘
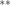
’ 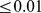, ‘
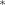
’ 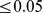 and ‘n.s.’ for non sognificant.

These results can appear as contradictory with our earlier result that dsCD4 does not exhibit phylogenetic signal. The reason for the effect of the phylogeny on regressions involving dsCD4 is that when the PCA is used to study trait correlations, the correction applied to the phylogeny (i.e. the value of 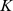 or 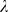) is calculated using the value of both traits (the model finds the covariance between trait values of pairs of tips that best explains the data assuming a Brownian model of evolution of the trait on the tree [26]).

## Discussion

The control of the virus genome on the virulence of an HIV infection is a controversial issue. HIV-1 is known to be more virulent than HIV-2. Within HIV-1 group M, there is also evidence that some subtypes are less virulent than others, e.g. viruses from subtype A seem to be associated with slower disease progression than viruses from subtype D [29]–[31]. Also, there is experimental evidence for differences in virulence among SIV viruses [32]. However, there is much less evidence supporting the existence of a control of the virus genotype over the duration of the infection within a given subtype of HIV-1. Here, we show that even within subtype B, the set-point viral load (spVL), which predicts HIV virulence, can be inherited from one infection to the next thus indicating that this trait depends strongly on the virus genotype. The validity of our approach is supported by the fact that we find heritability values close to 1 for a trait associated with drug resistance. Our results are statistically significant and robust to noise in the phylogeny. This robustness is important because HIV phylogenies tend to be inaccurate [22] due to processes such as recombination and co-infections.

The heritability value we find is high compared to previous studies [8]–[10], especially considering the fact that most (if not all) of these studies include hosts infected by different subtypes of HIV-1, which is likely to increase the genetical component of the variance observed in the population and hence the heritability. The magnitude of our estimate only compares with that of Hecht et al. [11]. However, one should be careful with comparing the two studies. First, they do not use spVL but the earliest measure of viral load. It is therefore possible that their measure is not linked to disease progression. Second, it is not clear if all the patients they consider are infected by the same subtype of HIV, which could increase the heritability, as explained above. Third, they include both treated and untreated patients, whereas we only include untreated patients. Fourth, and perhaps most important, they have only 49 patients in their largest dataset.

### Implications of virulence heritability

An increasing number of studies attempt to estimate heritability of HIV set-point viral load, which is a measure of virulence [6], [8]–[11]. The magnitude of the heritability value for spVL we find is of clinical and epidemiological importance.

The clinical importance stems from the fact that for a trait to be heritable from one infection to the next, there needs to be a control from the virus genotype over the trait. In the case of HIV, the course of the natural history of the infection is still largely not understood. For instance, the exact role of within-host evolution in the onset of AIDS is still unclear [33]. Here, we show that the duration of the infection is at least partially controlled by the genotype of the virus that infects the host. An implication is that some of the answers to AIDS pathogenesis might be found in the genome of the virus.

Trait heritability from one infection to the next also has implications for evolutionary epidemiology. For natural selection to act on a trait, three conditions must be fulfilled: the trait must be variable in the population, it must affect the fitness of individuals bearing it and it must be heritable (at least partially). This reasoning can be extended to evolutionary epidemiology by viewing an infection as an individual, and defining fitness as the number of new infections caused [34]. In the case of HIV, spVL is known to vary among patients [35] and to affect the infection fitness, because viral load correlates with the transmission rate [16], [36] and the virulence [16], [18], [21]. Our finding that spVL is highly heritable implies that this trait is subject to natural selection at the between-host level, which supports the possibility that a trade-off between virulence and transmission rate can drive the evolution of HIV [16]. This also sheds a new light on the ongoing debate concerning why studies have failed to provide a clear picture of how HIV virulence evolved over the last decade (some find that it decreased [37], [38], others find that it increased [17], [39]–[42] and some find no significant trend [43]–[49]). These conflicting trends could be explained by different dates of origin of the pandemics and different initial values of virulence [17]. Another possibility is that the host genotype control over virulence is greater than that of the virus (see next paragraph). Finally, the structure of sexual transmission networks is likely to have a strong effect on the fitness of an infection and hence on viral evolution.

### Host effects

Set-point viral load and disease progression have been shown to be affected by host factors, especially human leucocyte antigen (HLA) alleles [2], [3], [50], [51]. This could introduce a bias in our results if hosts with similar HLA alleles tend to be close in the transmission chain. Note that this bias also occurs in studies based on transmission pairs. Unfortunately, in the subset of the SHCS data we used, there was not enough information about the patients to correct for this bias. More generally, we cannot exclude correlations between the environments in which the patients live but these effects are likely to be smaller in our study than in studies based on known couples because the PCA does not require patient couples.

Another potential concern is that other diseases could be transmitted sexually with HIV that could affect trait values. The information of the SHCS allows us to show that there is no correlation between spVL and infection by hepatitis C, hepatitis B or syphilis (see Supplementary Results).

Furthermore, viruses evolving in similar within-host environment (either because of similar HLA alleles or drug treatments) might tend to evolve in a similar fashion. As a result, the proximity in the phylogeny could indicate proximity in within-host environment rather than proximity in the transmission chain. In order to limit the effect of host*virus interactions, we built a phylogeny using mutations on third codon positions only. As shown in the Supplementary Results, the outcome of the test is similar: 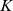 is equal to 0.52 for spVL and to 0.82 for prAZT in the MSM strict dataset. That a phylogeny built on synonymous mutations only leads to similar results allows us to rule out convergent evolution as a potential bias.

We do not know which proportion of the virus genotype effect is due to the interaction between virus and host genotypes (i.e. that some host genotypes are more sensitive to some virus genotypes). This implies that, in theory, the host control on spVL could also be high. A way to test this effect so that it could be compared to the present result would be to conduct a similar approach but with a host phylogeny instead of a virus phylogeny. The tip data would be the same and the results would tell us what the host genotype control over the trait is. This analysis could not be performed with the subset of the SHCS data we used in this study because we lacked information for many hosts. However, it might be achievable with another subset of the SHCS data.

More speculatively, we find that, in agreement with an earlier study [28], the correlation between spVL and dsCD4 is weak (low 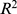). However, both these traits are known to be early predictors of virulence [18], [21]. A way to reconcile these two apparently contradictory facts would be to show that spVL and dsCD4 are both correlated with processes that add up to determine virulence. Another way to write this is that if 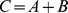, both 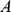 and 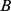 are correlated with 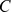 but 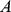 needs not to be correlated with 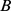. In our case, 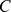 would be virulence and 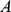 and 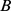 pathogenic processes linked with dsCD4 and spVL. We show that the virus has a strong control over spVL but not over dsCD4. If dsCD4 was shown to be strongly controlled by the host genotype, the 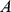 and 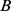 processes that contribute to virulence could be interpreted as a virus and a host contribution. Our results suggest that a way to better understand the progression to AIDS could be to disentangle host and viral contributions to virulence, which are highlighted here by the different control of the virus over dsCD4 and spVL.

### Advantages of the phylogenetic comparative approach

Some studies have used phylogenies of infections to understand the epidemiology of infectious diseases (see [52] and references therein). However, these studies are based on qualitative traits, usually the presence/absence at a given location (geographically or within a type of cells). Also, these studies use estimators to link the phylogeny and the qualitative traits that are different from phylogenetic signal and thus not directly associable with heritability.

To our knowledge, our study is the first application of the phylogenetic comparative approach (PCA) to infection quantitative traits. As such, it makes it relevant to discuss the prospects of its application to infectious diseases. The main advantage of the PCA is that it does not require any prior knowledge about the transmission pairs. First, this greatly increases the amount of data accessible. Indeed, in many cohort studies, such as the SHCS, there is no information about transmission pairs. Previous studies estimating the heritability of spVL have 155 [9], 194 [8], 112 [10] and 48 patients [11] in their largest dataset. Here, we have 661 patients in our largest dataset and 134 in the smallest. Having more patient data allows us to improve the phylogeny by focusing on MSM only. It also allows us to improve the estimation of spVL by selecting patients for whom we have several measures of viral load, which could explain why we detect higher signal than earlier studies in which many spVL had to be estimated from a single measurement [8]. Second, working without pairs reduces some of the biases inherent to studies based on known couples (e.g. living in the same environment). Third, it incorporates within-host evolution through the branch length. Even though all the genetic data we use originates from after the acute phase, the sampling time is indirectly taken into account because a virus sampled during chronic infection should generate longer branches in the phylogeny than a virus transmitted early in the infection.

The PCA can also be used to study correlations among infection traits. To our knowledge, no other study has raised the problem that, for rapidly evolving diseases, data from different patients are not independent and that regressions should be weighted by distance in the transmission chain.

Finally, for most non-human diseases, standard population genetics settings can be conducted to estimate experimentally trait heritability. In the case of human diseases, we work with epidemiological data. The PCA is thus especially suited to study rapidly evolving diseases of humans.

### Limitations

We were unable to detect phylogenetic signal for the log(spVL) when we used a liberal spVL definition or when we considered all the transmission groups. This is consistent with earlier studies that find that restricting the dataset to improve the phylogeny [8] or the spVL measure [10], [11] greatly increases the heritability estimate. Our interpretation is that the strict criterion we use allows us to improve the spVL measurement (thus decreasing measurement error) and that restricting the dataset to MSM patients yields a more accurate phylogeny (because of a better estimation of the transmission chain in the SHCS). Moreover, both restrictions remove the effect of confounding factors, such as patient sex or age, on trait values.

For completeness, we mention two alternative hypotheses to account for the low signal value in the ‘MSM liberal’ dataset. First, it is possible that high within-host variability in viral load measurements is positively correlated with the amount of within-host evolution, which itself has been shown to be linked with disease progression [53]. In short, our strict selection criterion might be selecting for infections with little within-host evolution. However, this is unlikely because, as we show in the Supplementary Results, the log(spVL) is slightly higher in the strict than in the liberal dataset. If variability in spVL was correlated with within-host evolution, we would expect patients from the strict dataset to have a slow disease progression (i.e. a low spVL). A possibility to test this effect would be to vary the level of strictness of the spVL definition. Second, it is possible that the method we use is less efficient at detecting phylogenetic signal in trees that are too large. In the Supplementary Results, we show that tree size could have a slight effect on the accuracy of 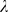, but this effect is less important than the intensity of the signal.

As shown in Figure 2, the correlation between heritability and phylogenetic signal is not 1 to 1. This means that, even if there is a strong control of the virus genotype on the spVL, it is not yet possible to say if this value is closer to 40 or 60%. Further analyses are required to understand analytically the exact mapping between heritability and phylogenetic signal in the case of infectious diseases.

Finally, we built the phylogeny using the *pol* gene, which is the gene routinely sequenced in the SHCS. However, using a different gene is unlikely to affect our results: a recent study shows that phylogenies built on clonal *env* sequences were almost identical to phylogenies built on *pol* sequences from known transmission pairs [54].

### Perspectives

The PCA argues that HIV set-point viral load is strongly controlled by the virus genotype. Our study can be extended by considering other infection life-history traits (e.g. the duration of the infection) or other infectious diseases that evolve rapidly enough in their host for a phylogeny to be inferred. Further studies are also needed to identify the specificities of the application of the comparative method to phylogenies of infectious diseases. Nevertheless, this approach opens new perspectives for evolutionary epidemiology by allowing a better understanding of how natural selection acts on infection traits. It also has the practical advantage to be applicable to many datasets of infectious diseases of humans because it does not require any prior knowledge of the transmission chain.

## Materials and Methods

### The data

The Swiss HIV Cohort Study (SHCS) is a nationwide prospective study based on voluntary participation of persons infected with HIV-1. The rationale, organisation and baseline characteristics of the study [15], [55] and the drug resistance database [56] we use have been described elsewhere in detail. Data of 1100 patients could be incorporated in this study. Each patient is represented only once in the data. We selected SHCS participants infected by HIV-1 subtype B (which is the majority in Switzerland) with a genotypic drug resistance test while still ART-naive and with at least three HIV RNA measurements. We only included measurements that were collected after the acute phase of HIV infection (as described in [25]), but prior to start of ART, or the first CDC C event, or the time when the CD4 count first drops below 200 cells. Many HIV-infected patients in Switzerland, as in other countries, receive their HIV diagnosis at a late disease stage and almost immediately start HIV treatment. However, these late presenting patients do not differ from other patients infected by HIV-1 subtype B [57], and this is not likely to introduce a bias on the 1100 patients we consider.

The three main transmission groups in the SHCS are heterosexuals (HET), injection drug users (IDU) and men having sex with men (MSM). We focused on MSM because, on the basis of all newly reported positive HIV Tests in Switzerland between 2000 and 2006 (Federal Office of Public Health, Switzerland), we estimate that the liberal dataset includes 25% of all newly diagnosed MSM, as opposed to 10% for IDU and 4% for HET. Also, a previous study has shown that patients from this transmission group tend to cluster in phylogenetic trees [25]. In our case, it means that the phylogeny we obtain using this transmission group only is likely to be closer to the actual transmission chain.

### The phylogenies

For each patient, we know the RNA sequence generated by bulk sequencing of the HIV polymerase (the *pol* gene). The sequence isolated from 49 patients infected by HIV-1 subtype C was used as an outgroup. We removed all major amino acid positions that are strongly correlated with antiretroviral drug resistance and built a different tree for each of the four subsets of the dataset. In all the trees we built, the ingroup and the outgroup were monophyletic. All phylogenies were built both with a maximum likelihood approach and with a Bayesian approach.

### The spVL

Measuring spVL often generates passionate debates. The notion of spVL originates from the realisation that during the asymptomatic phase of an HIV infection, the viral load remains generally stable. The problem is that fluctuations can occur in some patients [58]. Also, there seems to be a tendency for the viral load to increase during the asymptomatic phase [59], which means that defining spVL as a line (with a slope and an intercept) could provide us with more information. Overall, the most appropriate measurement for spVL largely depends on the data available and on the question asked.

Having multiple viral load measures in each patient allows to improve the quality of the spVL estimate. Some use the median value of viral load measurements [20]. Other studies on the host genetic control over spVL variations define spVL as the mean log10 virus load per mL in patients and only consider cases where all the viral load measurement fluctuate within a 0.5-log band around the patient specific mean [2], [3]. Our ‘strict’ definition of spVL is similar except for the fact that we allow measures to fluctuate in a 1-log band (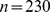). We also consider a ‘liberal’ definition, where spVL is the mean viral load taken over at least three consecutive viral loads, fluctuating within a 1-log band (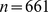). Note that spVL was a continuous (and normally distributed) trait in all our datasets so that the variation we observed are not likely to be linked to a specific allele of a gene.

The effects of patient age, sex and transmission group on the traits we study (spVL, dsCD4 and prAZT) are described in Supplementary Results (see also [19] for a review).

### The prAZT

This trait is linked to the probability that a virus is resistant to zidovudine (AZT) without ever having been exposed to this drug. It is estimated from the *pol* sequence using the geno2pheno system [60]. In order to remove potential correlations between this trait and the phylogeny, we only used the positions associated with drug resistance that we removed to build the phylogeny to estimate prAZT. These relevant positions were inserted in a neutral background sequence (for which there was no drug resistance).

### Phylogenetic signal

Phylogenetic signal measures the extent to which the fact that some species tend to have similar trait values can be explained statistically by their close evolutionary history (i.e. the fact that they share a recent common ancestor). Estimators are usually based on Felsenstein's method of independent contrasts [14], [24]. A contrast is the difference between two trait values of two tips of the phylogeny, which is weighted by the distance between the tips in the phylogeny. Mathematically, the contrast 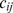 between two tips 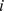 and 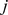 is given by:
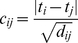
(1)where 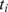 and 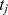 are the trait values of 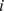 and 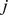 and 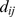 is the distance between 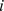 and 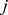 in the phylogeny.

If the variance in all the independent contrasts of a phylogeny is low (resp. high), it means that related species tend to have similar (resp. different) trait values. This simple approach is not entirely satisfactory because, for instance, it does consider the fact that specific shapes of the phylogeny can be more prone to exhibit higher or lower values in variance of contrasts. To solve this problem, we use two recently developed estimators.

The first estimator, 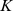 [12], is based on the mean squared error (MSE) of the contrasts or of the terms of Pagel's covariance matrix, which is described below. The ratio between the MSE obtained after transformation of the tree by a factor 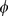 (denoted MSE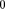) and the MSE obtained with the real tree (denoted MSE) indicates how well the transformed tree fits the data. 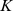 is a normalisation of this quantity. It is obtained by dividing the ratio MSE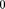/MSE by the expected ratio MSE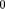/MSE given the shape and size of the phylogeny. 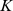 has the advantage of being normalised, i.e. that its value accounts for the fact that phylogenetic signal depends on the shape of the phylogeny. The significance of the estimate is assessed through a randomisation test based on a shuffling of the tip values.

The second estimator, 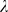, was introduced by Pagel [26], [61]. The idea is to formalise the phylogeny using a single variance-covariance matrix (denoted 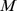), which predicts the covariance between the traits of two tips based on their distance in the phylogeny (i.e. how old their most recent common ancestor is) and assuming Brownian motion. From this covariance matrix 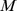, the model can generate a predicted distribution of traits (a vector 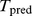) in the population (i.e. tip values). 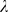 is used to multiply the off-diagonal terms of the matrix. It is then possible to generate a predicted distribution of tip values that depends on 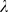 (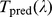). The phylogenetic signal is the value of 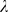 that generates the tip data (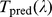) the closest to the observed data (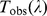). The fit is estimated with a Maximum Likelihood approach. 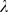 is less robust than 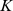 (see e.g. the variance in Figure 2), which is why we apply it to the set of trees resulting from a Bayesian estimation of the phylogeny and present the median value we obtain (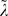). For further details about these estimators and about their high level of robustness, see the Supplementary Results.

### The model

We simulate an evolutionary process of a trait with known heritability values (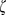) on a tree to compare the performance of our two estimators (Figure 2). We initiate the system with an ancestor that has a trait value (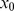) drawn from the empirical distribution of traits (spVL) in the population (note however that this model is more general and can be applied to any trait of any infectious disease). Every new infection is modelled as a branching in the tree: one of the new branches corresponds to the infecting individuals and is given the trait value of the ancestor branch (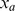), whereas the other branch corresponds to the infected individual and is given a new trait value (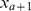). This new trait is obtained with the following rule:
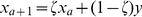
(2)where 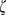 is the heritability of the trait and 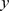 is a random variable drawn from the empirical trait distribution in the population (in the ‘liberal’ dataset). Another possibility would have been to model explicitly the environmental and the genetical component of the trait (as in [13]). The problem with such an approach is that it would require the introduction of a stabilising selection model, instead of the Brownian motion model, to describe trait evolution. Here, the 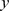 random variable is drawn in a trait distribution, which allows us to obtain a final set of trait values that has a mean and a variance close to what is observed empirically.

We introduce host death events as well as incomplete sampling of the hosts. The probability of dying was taken to be 1/3 of the probability of transmitting (thus approximating an 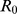 of 3 for the disease). We model the evolution over 13 generations and then sample uniformly at random 128 tips for each tree. There were 20 replicates for each of the heritability values we model. 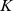 and 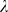 were estimated for each of the replicates. The resulting trees before sampling did not have the same size (because of the stochastic death process) but we found no significant effect of the intensity of sampling on the value of the estimator.

Further details about the Materials and Methods are available in the Supplementary Methods (Text S2).

### Ethics statement

The Swiss HIV Cohort Study (SHCS) has been approved by ethical committees of all participating institutions and written informed consent has been obtained from the participants. This project has been approved by the Scientific Board of the SHCS as project 606.

### Gene accession numbers

For scientific and ethical reasons explained in details in [25], only a fraction (approximately 10%) of the sequences of the SHCS is accessible via GenBank (accession numbers, GU344102GU344671). However, all data in the SHCS can be used for well-defined projects that are in accordance with the guidelines of the SHCS, if a corresponding project proposal is approved by the SHCS scientific board.

## Supporting Information

Text S1 Supplementary results.
(3.49 MB PDF)

Text S2 Supplementary methods.
(0.40 MB PDF)
