# Supplementary material for: Phylogenetic Approach Reveals That Virus Genotype Largely Determines HIV Set-Point Viral Load
Source: PLoS Pathog. 2010 Sep 30;6(9):e1001123. doi: 10.1371/journal.ppat.1001123 (PMC2947993; doi:10.1371/journal.ppat.1001123)
Supplement: Text S1 — Supplementary results. (3.49 MB PDF) [file ppat.1001123.s001.pdf]

Text S1: Supplementary Results to *Phylogenetic approach reveals that virus genotype largely determines HIV set-point viral load.*

Contents

A Significance of K values **2**

B Distribution of λ values **2**

C Robustness of tree to noise in the phylogeny **2**
- C.1 Summary figure . . . 2
- C.2 Heritability on bootstrapped trees . . . 5
- C.3 Adding errors to the tree . . . 5

D Signal in trees built with third codon positions only **6**

E Effect of the substitution model and branch swapping algorithm **7**

F Measuring heritability assuming stabilising selection **7**

G Effect of tree size on the estimator **7**

H Link between spVL and drug resistance **8**

I Confounding factors **8**
- I.1 Factors affecting spVL . . . 9
- I.2 Factors affecting dsCD4 . . . 10
- I.3 Factors affecting prAZT . . . 10

J Correlations between traits **12**

A Significance of K values

The significance of the values of the estimator for phylogenetic signal K is assessed through a randomisation procedure [1] (described in Text S2 'Supplementary Methods'). The p-values of this test are shown in Table S1. A non-significant p-value means that the signal found on the tree does not differ from what one would expect on any tree with a similar shape. Note that there is no p-value for the other estimator used in the main text (λ) because it is determined through a maximum likelihood approach. This is why we always calculate λ over many trees to increase the reliability of the measure (see Supplementary Methods).

Table S1. Phylogenetic signal (K) in the 4 datasets for three different traits. Here, as in all the tables, the significance code for the p-values is '***' ≤ 0.001, '**' ≤ 0.01, '*' ≤ 0.05, ^{a} is n.s. but ≤ 0.1 and ^{aa} is n.s. and > 0.1. Significant values are in bold font.

dataset	n	K for dsCD4	K for spVL	K for prAZT
MSM strict	134	0.45^{aa}	**0.59***	**0.91***
all strict	230	0.02^{aa}	0.03^{aa}	0.75^{aa}
MSM liberal	404	0.09^{a}	**0.09***	**0.82***
all liberal	661	0.002^{aa}	0.002^{aa}	**0.71***

B Distribution of λ values

In order to compensate for the variability in λ, we estimate it on 160 of the posterior trees (i.e. every 5th tree in the posterior) obtained with the Bayesian estimation of the phylogeny (see Supplementary Methods). Here, we show the distribution of these values of λ.

The tests for several of the trees failed to converge (i.e. $\lambda = 0$). However, we have reasons to think that this might not only be due to the shape of the phylogeny. First, because K estimated on the same trees revealed a significant signal. Second, because removing randomly 10% of the tips of a tree for which $\lambda = 0$ could lead to a tree with a $\lambda > 0$. This suggests that the problem could originate from the algorithm of the software we used. This is also why we only present the median value of λ estimated on many trees.

C Robustness of tree to noise in the phylogeny

C.1 Summary figure

A potential concern is that the heritability values we find might be limited because, as for most HIV studies [2,3], our phylogenies have a high level of uncertainty. We have no straightforward way to improve our phylogenies but it is quite simple to make them worse. We do this for the phylogeny obtained with the MSM strict dataset in two ways. First, we analyse the bootstrap trees of the maximum likelihood approach. Second, we introduce errors in the phylogeny by swapping a given number of tips randomly. The results are summarised in Figure S2 and further described below.

The rationale for these tests is that one might be concerned that we would get an even higher signal (especially for spVL) if our phylogeny was better. We show that making it worse does not affect the signal suggesting that the method is robust to noise in the phylogeny.

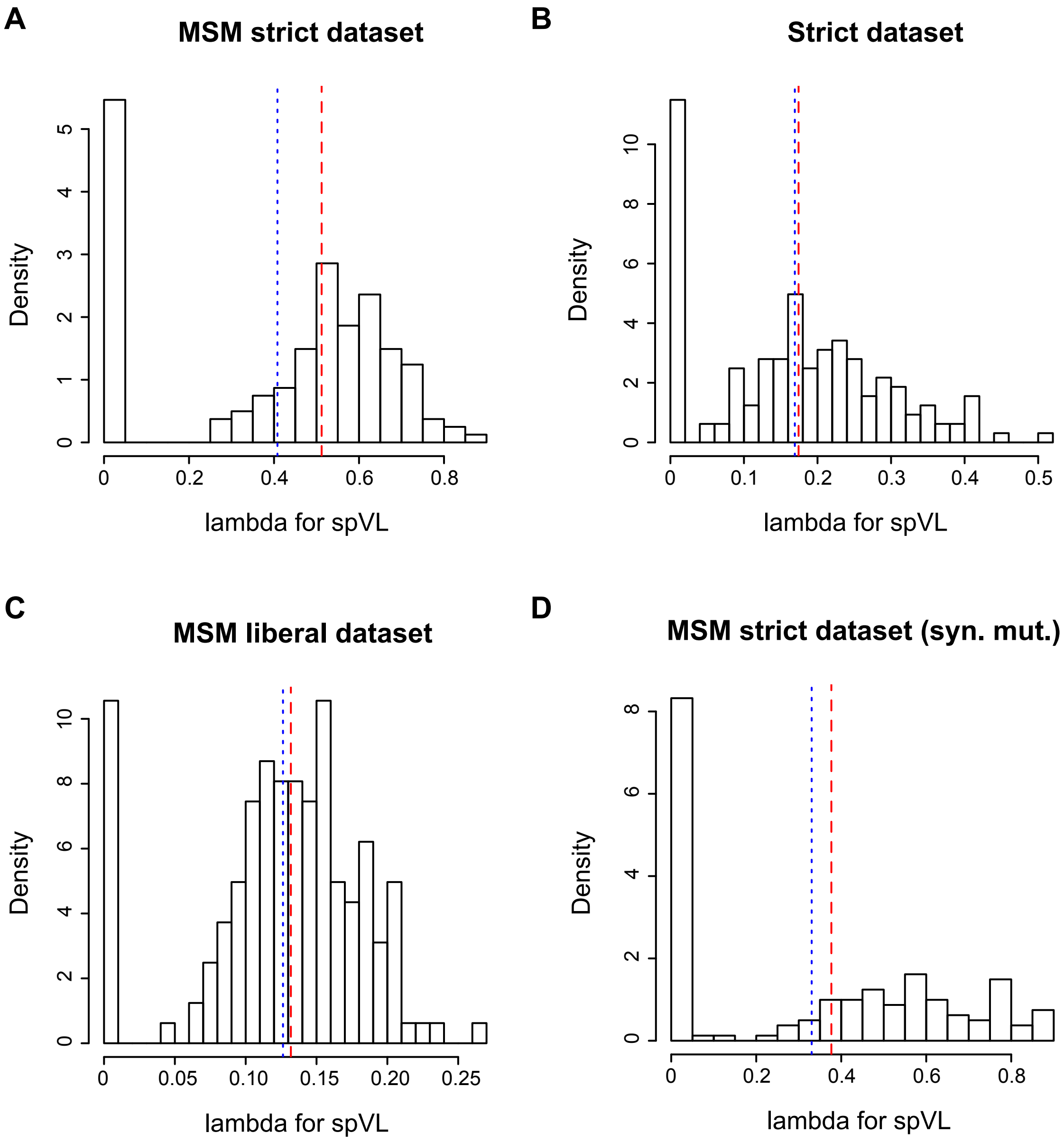

Figure S1. Distribution of the value of λ for the MSM strict (A), the strict (B), the MSM liberal (C) and the liberal dataset (D). The blue dashed line indicates the median and the red dotted line indicates the mean.

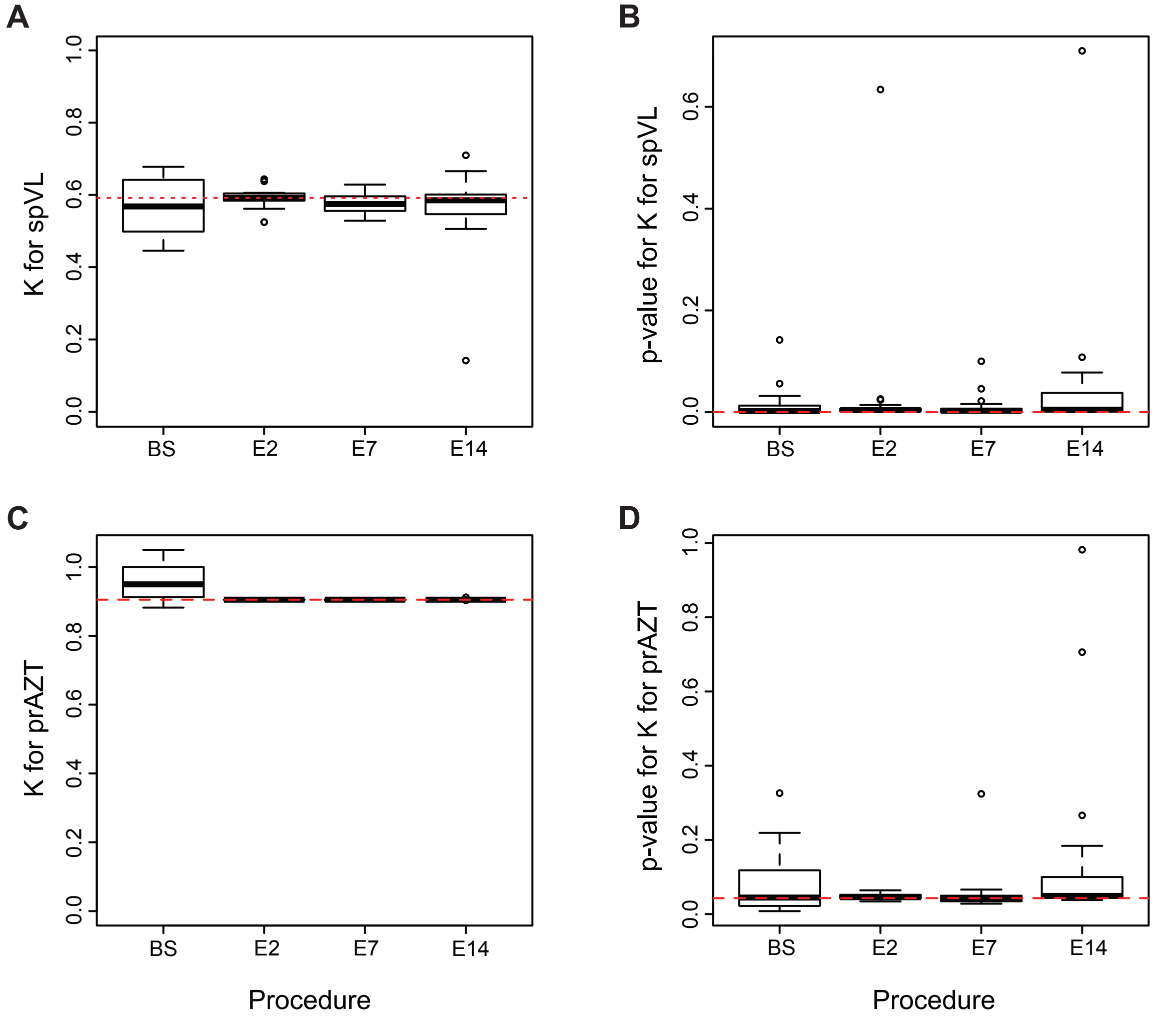

Figure S2. Robustness of the K estimator to errors and noise in the 'MSM strict' phylogeny. Panels A and C show phylogenetic signal value and panels B and D show the results of the randomisation tests. 'BS' indicates the value for bootstrapped trees and 'E2', 'E7' and 'E14' indicate the number of branch tips swapped in the procedure to generate errors (2, 7 or 14, which involves approximately 3%, 10% and 20% of the branches respectively). Each procedure was repeated 20 times. The red dashed line indicates the values found on the best maximum likelihood tree presented in the main text. The box plots show the median values, the three quartiles and the outliers. Only the significant values of K (with a p-value ≤ 0.05) are included in panels A and C.

C.2 Heritability on bootstrapped trees

We analysed the bootstrapped trees obtained with PhyML for the MSM strict dataset. All these trees are less accurate than the consensus tree because the bootstrap ignores the information of approximately $1/e$ of the nucleotides. K was calculated for 20 of these trees while λ was calculated for 100 trees. Table S2 shows the mean, median and standard deviation for both estimators.

Table S2. Phylogenetic signal in bootstrapped trees for the 'MSM strict' dataset. The standard deviation measures are the ones shown in the main text.

Estimator	trees	mean	standard deviation	median
K for spVL	17[1]	0.56	0.072	0.56
λ for spVL	100	0.48	0.24	0.40
K for prAZT	10[2]	0.96	0.055	0.95
λ for prAZT	100	0.99	0.32	1.06

[1] 4 trees were excluded from this analysis: two because their p-value was greater than 0.05 and two because they had unrealistically high values for K (1.42 and 2.52).

[2] 10 of the trees were excluded because they had a p-value greater than 0.05.

Note that for two of the bootstrapped trees, we found unrealistically high signal for spVL (K of 1.42 and 2.52). The value of λ estimated on these same trees was also high but more realistic (0.88 and 0.76). Finally, the value of K^*, which is another estimator introduced by Blomberg et al. [1] was of 0.66 and 0.61. Our interpretation is that bootstrapping generated these two trees with artificially high signal. We actually observed a similar effect when reducing the dataset to very low numbers ($n = 45$): most of the trees then yield non-significant signal but some trees can by chance lead to significant and very high signal. This underlines the importance of assessing phylogenetic signal with different approaches and testing for the robustness of the results.

We used the non-parametric Wilcoxon signed-rank test to check if the values we observe significantly differ from the values estimated on the best maximum likelihood tree (presented in Table 1 in the main text). We find that the bootstrapping has no effect on the estimates for spVL but the p-values of the randomisation tests tend to increase (Table S3). For prAZT, however, the value of K is tends to be significantly higher in the bootstrapped trees.

C.3 Adding errors to the tree

We swapped 2, 7 or 14 tips at random out of the 134 tips of the best MSM strict phylogeny obtained with PhyML. This corresponds to swapping approximately 3%, 10% and 20% of the tips. There were 20 repetitions for each procedure.

First, we found that adding errors has no effect on K for dsCD4. In other words, errors in the tree do not lead to false positive signal. Second, we found that trees that tend to lead to non-significant signal are associated with a value of K that deviates from the mean (either when K is unusually high or low). This could explain the robustness of the estimator: making the phylogeny worse thus affects the variance in the signal we observe but the mean value itself remains unaffected.

We apply the Wilcoxon test on the pooled error procedures (2, 7 or 14 branches swapped). Results are shown in table S4. We find that for both traits the value of K is not affected by the errors but that the p-value increases.

Table S3. Wilcoxon test for an effect of bootstrap trees of the 'MSM strict' dataset. V is the value of the test statistic. In all four cases, the alternative hypothesis is that the mean of the BS trees differs from the original mean. There are 20 replicates.

Test	true mean	V	p-value
K for spVL[1]	0.59	34	0.083
p-value for K spVL	0.0	78	*0.0024***
K for prAZT[2]	0.91	51	*0.014**
p-value for K prAZT	0.043	139	0.21

[1] 3 trees were excluded from this analysis: one because its p-value was 0.142 and two because they had unrealistically high values for K (1.42 and 2.52).
[2] 9 of the trees were excluded because they had a p-value greater than 0.05.

Table S4. Wilcoxon test for an effect of errors in the 'MSM strict' dataset. There are 60 repetitions for this procedure. V is the value of the test statistic. In all four cases, the alternative hypothesis is that the mean after sampling differs from the original mean.

Test	true mean	V	p-value
K for spVL[1]	0.59	504.5	0.094
p-value for K spVL	0.0	1081	*<0.001****
K for prAZT[2]	0.91	4	0.854
p-value for K prAZT	0.043	1267	*0.0015***

[1] 6 trees with p-values greater than 0.05 were excluded from the analysis.
[2] 18 trees with p-values greater than 0.05 were excluded from the analysis.

In addition to the Wilcoxon test, we also applied a generalised linear model (GLM) to see what the effect of increasing the amount of error is on our estimates. The GLM shows that increasing the number of errors does not affect the intensity of the average signal but that it significantly increases the p-values of the randomisation tests (Table S5).

D Signal in trees built with third codon positions only

We derived and analysed trees using third codon positions only to minimise potential convergent evolution due to drug resistance (Tables S6 and S7). This led to results qualitatively similar to that described in Table 1 in the main text.

Table S5. Generalised Linear Model for an effect of increasing number of errors in the 'MSM strict' phylogeny. All the K values included had a p-value lower than 0.05 for the randomisation test.

Test		Estimate	Std. Error	t-value	Pr(> |t|)
K for spVL	intercept	0.60	0.017	34.3	*<0.001******
	slope	-0.0027	0.002	-1.33	0.19
p-value for K spVL	slope	0.0039	0.0017	2.15	*0.036**
K for prAZT	intercept	0.91	0.00033	2720	*<0.001******
	slope	2e-5	3.7e-5	0.54	0.60
p-value for K prAZT	slope	0.01	0.0021	4.98	*<0.001******

Table S6. Phylogenetic signal (K) on trees built with third codon positions only.

dataset	n	K for dsCD4	K for spVL	K for prAZT
MSM strict	134	0.35^{aa}	**0.52*****	**0.86***
all strict	230	0.006^{aa}	0.008^{aa}	0.51^{11}

E Effect of the substitution model and branch swapping algorithm

Different substitution models can be used to build phylogenetic trees. We built a phylogeny from the 'MSM strict' dataset using a maximum likelihood approach with different models and estimated K for spVL. We found that the substitution model has little effect on the heritability value (Table S8).

We also built trees using the best of the NNI or SPR algorithm for branch swapping in PhyML (with a GTR substitution model). In this case, we found that $K = 0.585$ with a p-value of 4e-3 for spVL.

F Measuring heritability assuming stabilising selection

As explained in the Methods below, we used another estimator, d, which assumes a stabilising selection model of evolution instead of a Brownian model of evolution. As expected [1], we find similar qualitative values for d and K (Table S9). Quantitatively, the values are different but this is to be expected since d does not correct for the shape of the tree, which makes comparisons among trees more difficult.

G Effect of tree size on the estimator

In the main text, we mention the possibility that the value of our estimators for phylogenetic signal could be affected by tree size. To further test this assumption, we calculated λ for 80 trees of various sizes (average size was 274 tips with a standard deviation of 107) generated by the algorithm described in the Material and Methods of the main text. This allows us to simulate a process with known heritability. Here, we look at the percentage of change between the expected heritability measure and the measure observed. Using a generalised linear model, we look for the effect of tree size and of the known heritability value.

Table S7. Phylogenetic signal (λ) on trees built with third codon positions only. These values show the median value over 161 trees (see the Methods).

dataset	n	$\tilde{\lambda}$ for dsCD4	$\tilde{\lambda}$ for spVL	$\tilde{\lambda}$ for prAZT
MSM strict	134	0	0.38	1.05

Table S8. Phylogenetic signal in the strict datasets for spVL using K on a tree built with a specific substitution model.

Dataset	model	K for spVL
MSM strict	HKY85	0.56***
(n=134)	GTR	0.59***
	F84	0.57***
	TN93	0.56***
	K80	0.57***
	JC69	0.54***
	F81	0.54***

In table S10, we show that tree size does have a slight effect on the accuracy of λ (the larger the tree, the more accurate the estimate is). This increase might be due to the fact that larger trees also tend to exhibit an artificially high signal [1]. Here, this effect, however, is much smaller than the effect of the heritability value. If this value is too low, the accuracy of the estimation of λ decreases. This suggest that low heritability values might be the main constraint that prevent us from detecting signal in the 'MSM lib' and the 'all strict' datasets.

H Link between spVL and drug resistance

Some drug resistance mutations are known to have fitness costs and, since drug resistance is likely to be a highly heritable trait, one could legitimately be worried that the heritability we detect for spVL is a side effect of the heritability of prAZT. To rule out this effect, we looked for a correlation between drug resistance and spVL in our MSM strict dataset (and also in the liberal dataset). In this case, prAZT was determined on the whole *pol* sequence and not only on the few positions known to be associated with drug resistance because we were not using the sequences to build a phylogeny. We found no significant correlation between the two using a glm ($\Pr(> |t| = 0.28$, Figure S3). Therefore, it is very unlikely that heritability of spVL is due to variations in resistance to AZT (but other drug resistance mutations could have an effect).

Note that we did not use the phylogenetic comparative approach for this regression because prAZT is not a normally distributed trait.

I Confounding factors

As we show below in further details, the traits we measure, such as set-point viral load (spVL), can vary with patient age, sex or transmission group. These confounding factors are not specific to our dataset [4] and they are also observed in other cohorts than the SHCS (for a review, see [5]).

Table S9. Phylogenetic signal in the strict datasets for three different traits using d. The phylogenies of the 'liberal' datasets were too large for d to be computed.

dataset	n	d for dsCD4	d for spVL	d for prAZT
MSM strict	134	0^{aa}	0.72^{*}	1.01^{*}
all strict	230	0^{aa}	0.25^{***}	0.82^{*}

Table S10. Effect of tree size on the exactness of the value of λ. 20 phylogenies were simulated for 4 different heritability values (0.3, 0.5, 0.7 and 0.9) using the Method described in the main text.

Factor	Estimate	Std. Error	t value	$Pr(>\|t\|)$
intercept	1.07	0.21	5.12	$< 0.001^{***}$
**size**	-0.0016	0.0007	-2.18	0.033^{*}
**known heritability**	-1.14	0.30	-3.83	$< 0.001^{***}$
size * heritability	0.0021	0.0011	1.96	0.054

Confounding factors can affect our estimate of heritability because, for instance, patients from the same transmission group have been shown to cluster on the phylogeny [6]. Figure S4A shows that, in the SHCS, men having sex with men (MSM), are significantly older than heterosexuals (HET), who are older than intravenous drug users (IDU). Also, as shown on Figure S4B, set-point viral load is significantly lower for heterosexuals. Men in the SHCS tend to be older than women (Figure S4C) and to have a higher spVL (Figure S4D). Overall, spVL tends to increase with age in the liberal dataset (Figure S4E in red) but not when the dataset is narrowed to MSM or to patients fitting the strict spVL criterion. Finally, the largest proportion of the patients included in this study are MSM (Figure S4F), which reflects the structure of the SHCS [4].

I.1 Factors affecting spVL

In our largest dataset, spVL is affected by patient sex (males tend to have a higher spVL), age (older patients have higher spVL) and transmission group (MSM and IDU have higher spVL than HET). Finally, the selection criterion that we use to minimise within-host patient variance in viral load also affects spVL (this criterion tends to remove patients with a spVL lower than the average). This is summarised in Table S11.

When we only consider the MSM transmission group (Table S12), we find that spVL is mainly affected by the spVL selection criterion and the patient age. Again, MSM patients with less variance in viral load tend to have slightly higher spVL.

If we consider the 'strict' dataset (Table S13), we find no significant effect on spVL. This suggest that the improvement in detection of spVL heritability in the MSM strict dataset is due to the phylogeny.

Finally, in the 'MSM strict' dataset (Table S14), none of the factors influences spVL.

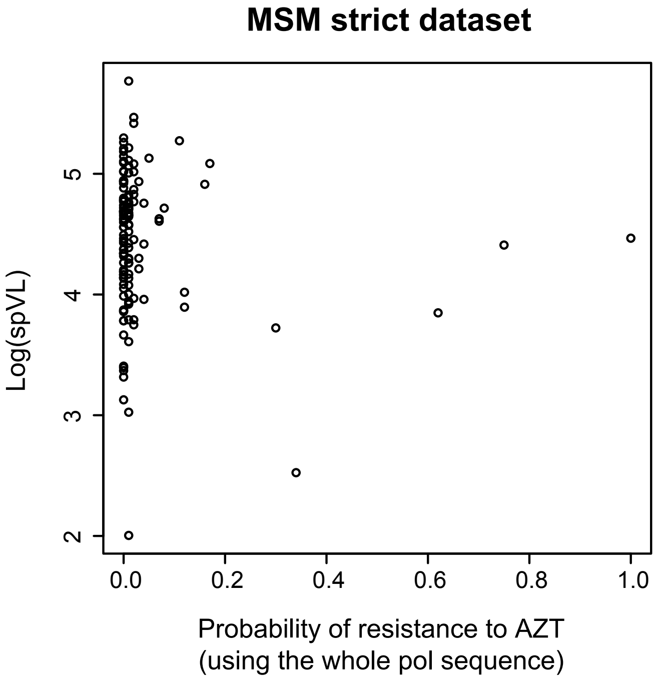

Figure S3. spVL as a function of prAZT in the MSM strict dataset.

I.2 Factors affecting dsCD4

If we apply a similar approach to dsCD4 (Table S15), we find that this trait is strongly affected by the transmission group (IDU have a lower dsCD4). Note that the confounding factor comes from men in the IDU transmission group. Also, co-infection by hepatitis C can affect dsCD4 in IDU.

In the MSM strict dataset (Table S16), we only get biases linked with STDs (syphilis or HBV). This means that phylogenetic signal for dsCD4 could be overestimated. However, we do not detect any signal (see the main text).

I.3 Factors affecting prAZT

If we apply a similar approach to prAZT (Table S17), we find that, as dsCD4, this trait is strongly affected by the transmission group (IDU tend to be more resistant to AZT).

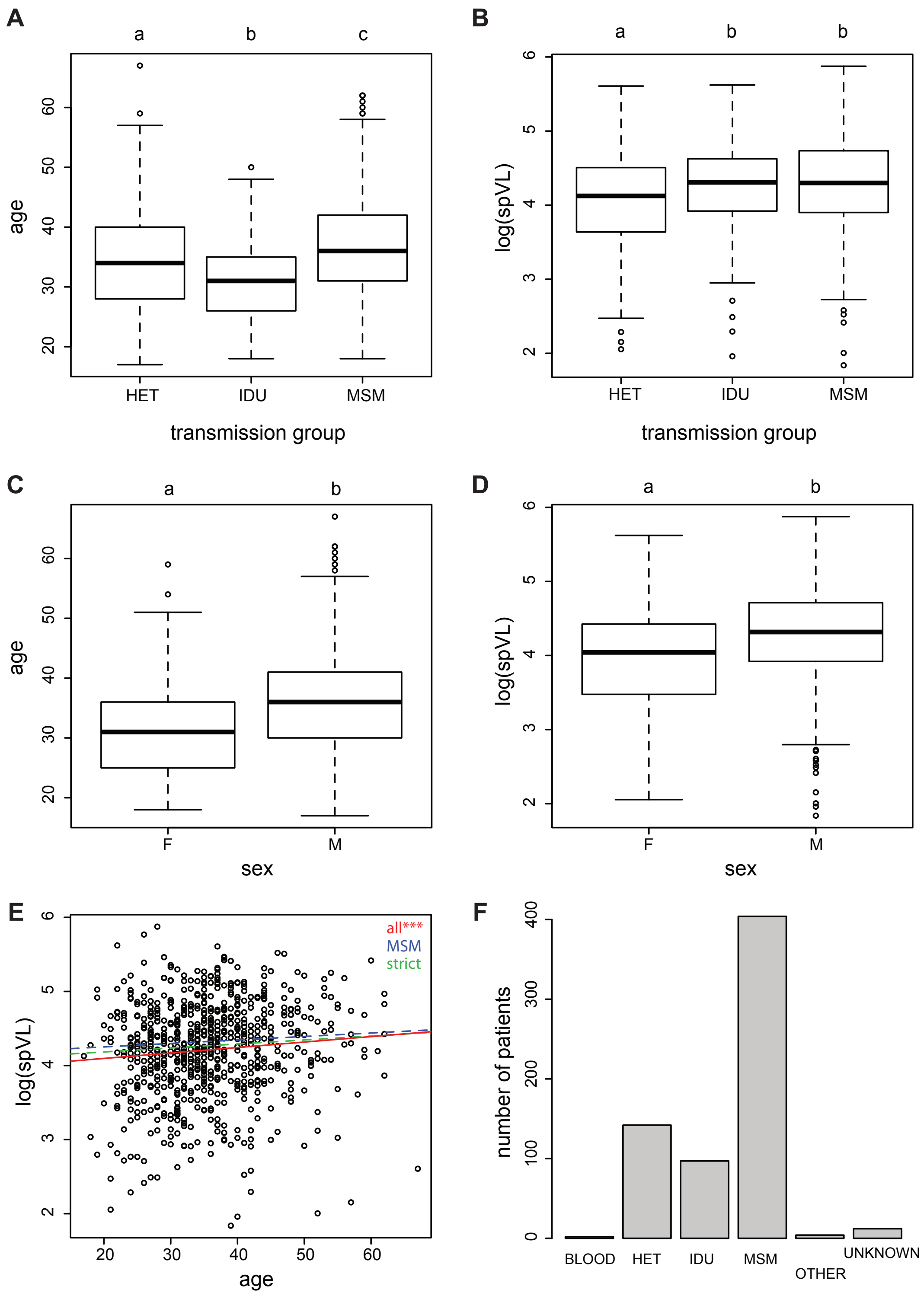

Figure S4. Age (A, C and E), sex (C and D), spVL (B, D and E) and transmission group (A, B and F) in the liberal dataset. Regression lines in panel E are obtained with a glm for the liberal dataset (in red, p-values< 0.001), the MSM liberal (in blue, n.s.) and the strict dataset (in green, n.s.).

Table S11. Effect of sex, age, STD, transmission group and spVL criterion on spVL. We use a generalised linear model (GLM) on the liberal dataset ($n = 661$ patients) with 7 factors (sex, age, HCV, HBV, syphilis, transmission group and spVL selection criterion). We included all the interactions between factors in the test but we only show significant interactions.

Factor	Estimate	Std. Error	t value	$\Pr(>\|t\|)$
intercept	3.03	0.36	8.4	<0.001***
**sex** (M)	1.77	0.58	3.06	0.0023**
**age**	0.0229	0.011	2.11	0.035*
HCV	1.15	2.35	0.49	0.62
HBV	0.58	3.08	0.19	0.85
syphilis	0.08	50.1	0.002	0.99
**transmission group (MSM)**	-1.07	0.51	-2.1	0.037*
transmission group (IDU)	-1.33	1.38	-0.96	0.34
**strict criterion**	0.20	0.68	0.30	0.77
**age * sex (M)**	-0.039	0.016	-2.45	0.0145*
**age * transmission group (MSM)**	0.029	0.013	2.19	0.0293*

Table S12. Effect of age, STD and spVL criterion on spVL for the 'MSM liberal' dataset. Here $n = 404$ patients and there are 5 factors (age, HCV, HBV, syphilis and spVL selection criterion).

Factor	Estimate	Std. Error	t value	$\Pr(>\|t\|)$
intercept	3.73	0.23	16.0	< 0.001***
**age**	0.013	0.0064	2.06	0.04*
HCV	0.77	1.96	0.39	0.69
HBV	0.36	0.40	0.90	0.37
syphilis	1.00	1.02	0.98	0.33
**strict criterion**	0.96	0.36	2.66	0.0082**
**age * strict**	-0.019	0.0098	-1.97	0.05*

J Correlations between traits

To study correlations between traits with the comparative approach we make some assumptions about the trait distributions [7]. Here, spVL and dsCD4 are normally distributed in the population, as shown on Figure S5. The prAZT distribution was strongly biased towards 0, which could alter the correlations involving this trait. This is why we ignored this trait in the analyses. Table S18 summarises all the correlations we tested in our datasets using different methods to correct for phylogenetic dependence. All the correlations involving the CD4 were estimated using only patients with at least 5 CD4 counts during the asymptomatic phase. The sample sizes were 65, 99, 304 and 478 instead of 134, 230, 404 and 661.

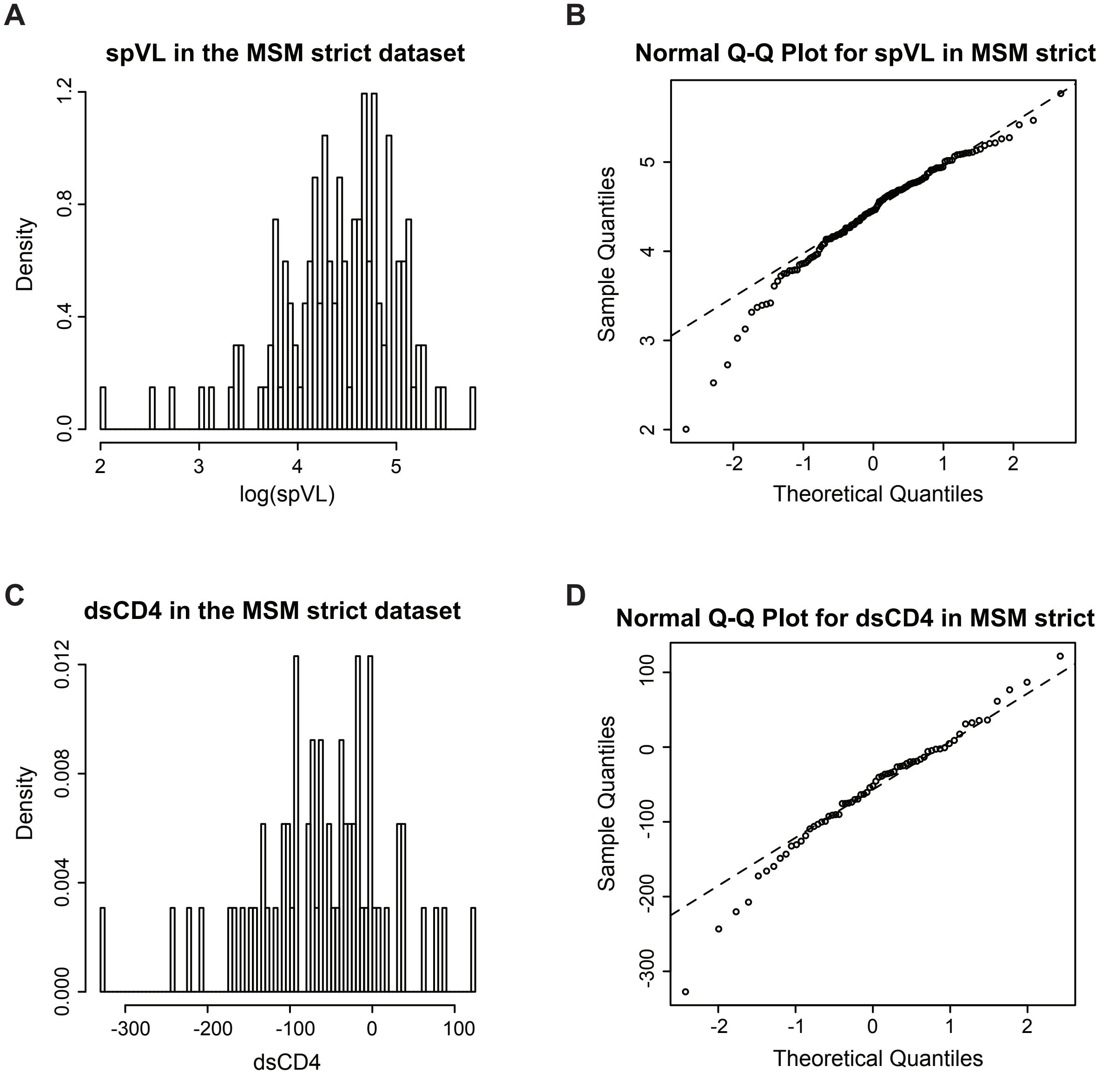

Figure S5. Distribution of spVL (A) and dsCD4 (C) in the MSM strict dataset. Panels B and D are normal quantile-quantile plots, where the dashes line passes through the first and third quartiles. A plot close to the line indicates a normal distribution.

Table S13. Effect of sex, age, STD and transmission group on spVL for the 'strict' dataset. Here $n = 230$ patients and there are 6 factors (sex, age, HCV, HBV, syphilis and transmission group).

Factor	Estimate	Std. Error	t value	Pr(> $\|t\|$)
intercept	3.23	0.54	5.98	<0.001***
sex (M)	0.48	1.04	0.46	0.64
age	0.021	0.017	1.21	0.23
HCV	2.28	2.66	0.86	0.39
HBV	-1.04	2.12	-0.49	0.62
syphilis	-0.95	2.04	-0.47	0.64
transmission group (IDU)	-0.97	3.02	-0.32	0.75
transmission group (MSM)	0.98	0.93	1.05	0.3

Table S14. Effect of age and STD on spVL for the 'MSM strict' dataset. Here $n = 134$ patients and there are 4 factors (age, HCV, HBV and syphilis).

Factor	Estimate	Std. Error	t value	Pr(> $\|t\|$)
intercept	4.17	0.23	18.2	<0.001***
age	0.0053	0.0063	0.85	0.40
HCV	0.30	0.54	0.55	0.59
HBV	-0.02	0.47	-0.047	0.96
syphilis	0.092	0.87	0.11	0.92

Table S15. Effect of sex, age, STD, transmission group and spVL criterion on dsCD4. We use a GLM on the liberal dataset ($n = 661$ patients) with 7 factors (sex, age, HCV, HBV, syphilis, transmission group and spVL selection criterion).

Factor	Estimate	Std. Error	t value	Pr(> $\|t\|$)
intercept	-13.7	76.4	-0.18	0.86
sex (M)	-76.7	119	-0.64	0.52
age	-1.35	2.34	-0.58	0.56
HCV	107.4	450	0.24	0.81
HBV	-162	588	-0.28	0.78
syphilis	-8163	9547	-0.86	0.39
transmission group (IDU)	137.3	325	0.42	0.67
transmission group (MSM)	34.1	103.8	0.33	0.74
strict criterion	-71.8	148.2	-0.48	0.63
**sex (M) * IDU**	5778	1781	3.25	0.0013**
**sex (M) * age * IDU**	-201.9	58.2	-3.47	0.00058***
**sex (M) * HCV * IDU**	-5428	1923	-2.82	0.0050**
**sex (M) * strict criterion * IDU**	729.8	300.5	2.43	0.016*
**sex (M) * age * HCV * IDU**	191.5	62.7	3.06	0.0024**

Table S16. Effect of sex, age, STD, transmission group and spVL criterion on dsCD4 in the MSM strict dataset.

Factor	Estimate	Std. Error	t value	Pr(> $\|t\|$)
intercept	-46.6	38.1	-1.22	0.22
age	-0.31	1.09	-0.29	0.78
HCV	-150.6	127.5	-1.18	0.24
HBV	107.6	95.7	1.12	0.26
syphilis	-477.1	152.3	-3.13	0.0023**
**age * syphilis**	13.2	4.26	3.10	0.0025**
**sex (M) * HCV * IDU**	-5428	1923	-2.82	0.0050**
**HBV * syphilis**	-265.8	105.0	-2.53	0.013*

Table S17. Effect of sex, age, STD, transmission group and spVL criterion on prAZT. We use a GLM on the liberal dataset ($n = 661$ patients) with 7 factors (sex, age, HCV, HBV, syphilis, transmission group and spVL selection criterion).

Factor	Estimate	Std. Error	t value	Pr(> $\|t\|$)
intercept	1.2e-3	7.6e-2	0.016	0.99
sex (M)	7.4e-2	0.12	0.61	0.54
age	-2.4e-5	2.3e-3	-0.011	0.99
HCV	-1.2e-3	4.9e-1	-0.002	0.99
HBV	-1.2e-3	4.4e-1	-0.002	0.99
syphilis	-0.49	10.5	-0.047	0.96
**transmission group (IDU)**	1.32	0.29	4.57	<0.0001***
transmission group (MSM)	-0.044	0.11	-0.41	0.68
strict criterion	-1.2e-3	0.14	-0.008	0.99
**sex (M) * IDU**	-1.4	0.66	-2.13	0.034*
**age * IDU**	-0.029	8.1e-3	-3.57	0.00039***
**strict criterion * MSM**	-0.59	0.24	-2.52	0.012*
**age * strict criterion * MSM**	0.014	6.2e-3	2.3	0.022*

Table S18. Regression between traits with or without correction for phylogenetic signal. OLS is the ordinary generalised least square (using the gls tool in R), RegBM indicates a correction based on the tree assuming brownian motion [8, 9], RegP indicates a correction based on the tree using Pagel's λ [10] and RegBM indicates a correction following [11]. For RegP and RegBM the parameter describing the amount of signal was assumed to be 1 but the value has little effect on the results.

Traits	Data set	Slope (SE)	Y-intercept (SE)	ln(likelihood)	AIC
spVL vs. dsCD4	*MSM strict*				
	OLS	-1.7e-3*(8.1e-4)	4.26***(0.08)	-56	119
	RegBM	-2.7e-3***(6.8e-4)	4.1***(0.34)	-57	121
	RegP	-1.9e-3*(7.6e-4)	4.23***(0.25)	-55	117
	RegG	-2.6e-3***(6.8e-4)	4.13***(0.3)		
	all strict				
	OLS	-2.1e-3**(6.9e-4)	4.1***(0.07)	-89	184
	RegBM	-2.6e-3***(6.2e-4)	4.0***(0.32)	-93	192
	RegP	-2.0e-3**(6.8e-4))	4.1***(0.14)	-89	186
	RegG	-2.3e-3***(6.2e-4)	4.2***(0.31)		
	MSM liberal				
	OLS	-1.8e-3***(4.3e-4)	4.1***(0.04)	-288	583
	RegBM	-6.3e-3***(1.7e-4)	3.0***(0.66)	-478	963
	RegP	-1.8e-3***(4.3e-4)	4.1***(0.11)	-288	584
	RegG	-6.0e-3***(2.0e-4)	3.5***(0.77)		
	all liberal				
	OLS	-1.8e-3***(3.6e-4)	4.1***(0.04)	-476	958
	RegBM	-5.3e-3***(1.2e-4)	3.6*(1.8)	-1327	2659
	RegP	-1.7e-3***(3.6e-4)	4.0***(0.14)	-473	955
	RegG	-5.3e-3***(1.3e-4)	3.6^{aa}(2.3)		
Variation of trait values among risk groups	*all strict*				
	OLS	spVL*** and dsCD4***			
	RegBM	spVL*** and dsCD4^{aa}			
	RegP	spVL*** and dsCD4***			
	all liberal				
	OLS	spVL*** and dsCD4***			
	RegBM	spVL*** and dsCD4^{aa}			
	RegP	spVL*** and dsCD4***			

References

1. Blomberg SP, Garland TJ, Ives AR (2003) Testing for phylogenetic signal in comparative data: behavioral traits are more labile. Evolution 57: 717–745.

2. Leitner T, Escanilla D, Franzen C, Uhlen M, Albert J (1996) Accurate reconstruction of a known HIV-1 transmission history by phylogenetic tree analysis. Proc Natl Acad Sci USA 93: 10864–10869.

3. Hué S, Clewley JP, Cane PA, Pillay D (2004) HIV-1 pol gene variation is sufficient for reconstruction of transmissions in the era of antiretroviral therapy. AIDS 18: 719–728.

4. The Swiss HIV Cohort Study (2010) Cohort Profile: The Swiss HIV Cohort Study. Int J Epidemiol in press: doi: 10.1093/ije/dyp321.

5. Langford SE, Ananworanich J, Cooper DA (2007) Predictors of disease progression in HIV infection: a review. AIDS Res Ther 4: 11.

6. Kouyos RD, von Wyl V, Yerly S, Böni J, Taffé P, et al. (2010) Molecular epidemiology reveals long-term changes in HIV type 1 subtype B transmission in Switzerland. J Infect Dis 201: 1488-97.

7. Freckleton RP (2009) The seven deadly sins of comparative analysis. J Evol Biol 22: 1367-75.

8. Felsenstein J (1985) Phylogenies and the Comparative Method. Am Nat 125: 1–15.

9. Martins EP, Hansen TF (1997) Phylogenies and the comparative method: A general approach to incorporating phylogenetic information into the analysis of interspecific data. Am Nat 149: 646–667.

10. Freckleton RP, Harvey PH, Pagel M (2002) Phylogenetic analysis and comparative data: a test and review of evidence. Am Nat 160: 712–726.

11. Garland Jr T, Ives AR (2000) Using the past to predict the present: confidence intervals for regression equations in phylogenetic comparative methods. Am Nat 155: 346–364.
