# Supplementary material for: Phylogenetic Approach Reveals That Virus Genotype Largely Determines HIV Set-Point Viral Load
Source: PLoS Pathog. 2010 Sep 30;6(9):e1001123. doi: 10.1371/journal.ppat.1001123 (PMC2947993; doi:10.1371/journal.ppat.1001123)
Supplement: Text S2 — Supplementary methods. (0.40 MB PDF) [file ppat.1001123.s002.pdf]

Text S2: Supplementary Methods to *Phylogenetic approach reveals that virus genotype largely determines HIV set-point viral load.*

Contents

A About the data **2**

B Building the phylogenies **3**

C Estimating prAZT **4**

D The phylogenetic comparative approach **5**

D.1 Felsenstein's independent contrasts 5

D.2 Estimators of Blomberg et al. 5

D.3 Estimators of Pagel and Lynch 5

E Estimating correlations between traits **6**

A About the data

The Swiss HIV Cohort Study (SHCS) is a nationwide prospective study based on voluntary participation of persons infected with HIV-1. As of May 2009, a total of 15694 patients have been enrolled in the SHCS but the amount of information known varies across patient and only 1100 patients could be incorporated in this study. The rationale, organisation and baseline characteristics of the study [1–3] and the drug resistance database [4] have been described elsewhere in detail and a continuously updated description can be found at www.shcs.ch.

We selected SHCS participants infected by HIV-1 subtype B with a genotypic drug resistance test while still ART-naive and with at least three HIV RNA measurements. This subtype is the majority in Switzerland [5] and in Europe [6]. This restricted the available dataset to 1100 patients, mainly because of inadequate numbers of pre-ART HIV RNA measurements to become eligible for this analysis. Many HIV-infected patients in Switzerland, as in other countries, receive their HIV diagnosis at a late disease stage and almost immediately start HIV treatment. However, these late presenting patients do not differ from other patients infected by HIV-1 subtype B [7], and this is not likely to introduce a bias on the data we analyse.

We want to measure the set-point viral load (spVL), here defined as the mean viral load value during the asymptomatic phase of an HIV infection. This is why we only included measurements that were collected after the acute phase of HIV infection (i.e. after more than 180 days since the infection, where the date of infection is estimated as described in [8] and [5]), but prior to start of ART, or the first CDC C event, or the time when the CD4 count first drops below 200 cells (whichever occurred first). Also, we imposed these measures to be done over a maximum time span of three years from the first eligible HIV RNA. Finally, measurement intervals had to be greater than 60 days.

Most studies on heritability of HIV have limited patient data to estimate set-point viral load. In the Rakai cohort, for instance, spVL is inferred in many patients from a single viral load measurement [9, 10]. Having at least three viral load estimates per patient allowed us to see that off-treatment HIV RNA measurements demonstrated a high variability in most patients. High variability in viral load measurements can be interesting in itself but it decreases the accuracy of the set-point value estimate. In order to select cases where there is a set-point viral load to measure, we applied two algorithms. For the first algorithm (Figure S6A), we followed previous studies [11, 12] and selected patients whose HIV RNA fluctuated within a 1 log band around the patient specific mean (the 'strict' definition, $n = 230$ patients). Here, we also introduced a second algorithm (Figure S6B), where the set-point was estimated as the mean viral load taken over at least three consecutive and 'stable' HIV RNA, fluctuating within an 1 log band from one HIV RNA to the next (the 'liberal' definition, $n = 661$ patients). The 1 log band still allows for important changes in viral load and thus to include patients in whom the viral load increases.

These algorithms ensured that only patients with low HIV RNA variability were selected and thus for whom the average of all off-treatment viral loads most likely reflects an accurate estimate of set-point viral load (see also, [11, 12]). Of course, this, as any process data selection, could introduce a bias in the results (see below). However, there is no straightforward explanation as to how decreasing within-host variance could artificially increase spVL heritability. The main effect we see is that confounding factors on spVL are removed when we consider the strict dataset.

Concerning the decline slope of the CD4 T-cells (dsCD4), we also investigated a dataset where we use at least 5 measurements of CD4 to estimate the slope (instead of 3), but this did not affect the results.

Finally, for both datasets, we create a sub-dataset by studying patients from the 'men having sex with men' (MSM) transmission group separately. The reason for this is twofold. First, this is the transmission group for which representativeness of our dataset is highest (see the main text). Second, when analysing the data, we found that sex, and in a lower extend transmission group and age, had an effect on spVL (see section **??** for a detailed analysis). When restricting our dataset to the MSM only, these factors do not have a significant effect anymore. dsCD4 was affected by transmission group and by infection by

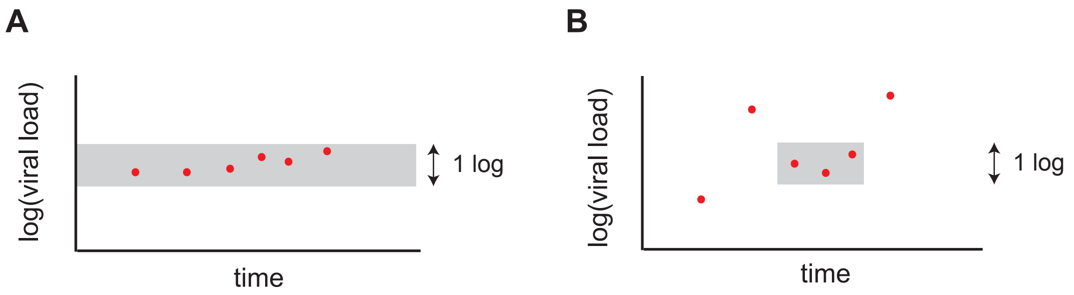

Figure S6. Illustration of the criterion used to select patient in the 'strict' (A) and in the 'liberal' (B) case. Red dots indicate RNA measurements done in a patient. Panels A and B correspond to different patients. The grey strip has a width of 1 log. In the 'strict' case, all the measurements have to be in the strip whereas in the 'liberal' case only three consecutive measurement need to be in a strip. Patients A and B can both be included in the 'liberal' dataset but only patient A can be included in the 'strict' dataset.

hepatitis C virus (HCV). These effects are absent when we focus on MSM only.

A legitimate concern is that heritability could be affected by multiple infections: if some of the patients harbour two sexually transmitted diseases (STD) and if the interaction between the two affects the trait value, this could increase the intensity of the signal we measure. The SHCS includes information about three other diseases that can be transmitted sexually: hepatitis C virus (HCV), hepatitis B virus (HCV) and syphilis. We found no significant correlation between spVL and any of these STD. This corroborates previous studies showing that co-infections by HIV and HCV have no effect on the morbidity due to HIV [13]. Some STD had a significant effect on dsCD4. Note that this should then tend to artificially increase phylogenetic signal for this trait but that we never find signal for dsCD4, suggesting that if there is any bias, it is weak. Further details about these analyses can be found above in section **??**.

B Building the phylogenies

For each patient, we know the RNA sequence data of the HIV polymerase (the *pol* gene). We included the RNA isolated from 49 patients infected by a different subtype of HIV (C instead of B) as an outgroup. We removed all major amino acid positions that are strongly correlated with antiretroviral drug resistance according to the International AIDS Society - USA (IAS-USA) guidelines [14] (positions 30, 32, 33, 46, 47, 48, 50, 54, 76, 82, 84, 88, and 90 in the protease and 41, 62, 65, 67, 69, 70, 74, 75, 77, 100, 103, 106, 108, 115, 116, 151, 181, 184, 188, 190, 210, 215, 219, 225, and 236 in the reverse transcriptase). We built a different tree for each of the four subsets of the dataset. In all the trees we built, the outgroup was monophyletic.

We also built trees using third codon positions only. The reason for these additional trees is that we wanted to decrease the likeliness that sequence similarity is due to selection (convergent evolution) rather than common descent. Convergent evolution can occur for drug resistance and lead to clustering in the tree that is not related to a common transmission history [15].

All the data manipulation and tree plotting was done using the 'ape' package in R ([16], freely available at http://www.R-project.org/). For further details, see [17].

The first method to construct a phylogenetic tree is based on a maximum likelihood estimate, which is appropriate for large phylogenies. It was performed using the software PhyML 3.0.1 [18]. The model

of nucleotide substitution was chosen to be the general time reversible (GTR + I + Γ) model with four Gamma rate categories. In the Text S1, we show that other substitution models or more complex branch swapping processes (SPR instead of NNI) lead to similar results.

The second method is based on a Bayesian estimation of the phylogeny. A Bayesian tree inference method looks through tree space and includes the highly supported trees into the posterior, i.e. the output is not a single tree but a set of trees. It is performed using the software BEAST v1.4.8 [19]. The model of nucleotide substitution was chosen to be the general time reversible (GTR + I + Γ) model with four Gamma rate categories. Base frequencies were estimated empirically. Rates were allowed to change according to a relaxed clock model with an uncorrelated lognormal prior. The prior on the trees was chosen to be the constant rate birth-death model. We ran the chain for 100 million generations and sampled every 100 000 tree. Convergence was checked with Tracer 1.4.1. The first 20 per cent of the trees was eliminated as burn-in. Effective sampling sizes were at least 146 for 'MSM strict', 97 for 'strict', 5.4 (which means no convergence) for 'MSM liberal' and 137 for 'MSM strict' based on third position codons only (but the effective sampling sizes were much higher for most parameters). Note that the posterior of the 'MSM liberal' dataset did not converge due to the big dataset (analysis took several weeks). For the other datasets, the convergence criteria were met.

Finally we analysed 160 of the posterior trees in each datasets (i.e. every 5th tree in the posterior). The analysis we did on the posterior sample showed the same trend as the analysis on the ML tree.

C Estimating prAZT

In order to check for the consistency of our method, we wanted to measure the heritability for a trait, which we expected to be heritable. Drug resistance is a good candidate because, in HIV, it is known to be associated with the virus genotype. If we know the *pol* sequence, it is even possible to estimate the probability that a patient will develop resistance to zidovudine (AZT) if treated with this drug. This is done using the geno2pheno tool (www.geno2pheno.org, [20]). The advantage of using probability scores is that it allows comparisons among patients.

The problem we faced is that since the estimate of the probability of resistance (prAZT) is based on the virus RNA, there could be a correlation between the phylogeny and the trait. Such a correlation is a problem because it will increase the heritability of the trait. To solve this problem, we based the estimate of prAZT *only* on the amino acid positions that are strongly correlated with antiretroviral drug resistance (see above and [14]), which we removed to build the phylogeny. For the other positions, we used the same sequence, which was associated with a 0 probability of resistance to AZT. This allowed us to have a measure of prAZT, which is at the same time independent from the phylogeny and expected to be highly heritable.

Because of our large sample size, we could not use the web interface directly and the estimation for our 661 patients was conducted by one of the researchers involved in the geno2pheno project (Dr. Joachim Büch).

Note that the patients included in our dataset are all untreated, therefore, any positive value of prAZT is has been selected in a previous host (especially knowing that transmitted AZT mutations tend to have a low fitness impact on the virus and tend to persist for several years). This is why prAZT is a trait linked to the probability that a virus is resistant to AZT without ever having been exposed to this drug.

The values of prAZT were affected by patient transmission group and slightly by HCV co-infection. As for dsCD4, these effects disappear when restricting our dataset to the MSM.

D The phylogenetic comparative approach

D.1 Felsenstein's independent contrasts

Felseinstein's original idea is that phylogenies can be used to estimate whether species that are close in the tree tend to have similar trait values [15,21]. He derived an approach known as the independent contrast. A contrast is a measure between two taxa of the phylogeny that corresponds to the difference in their trait value weighted by their distance in the tree. By estimating the variance of all the independent contrasts for a given trait on a tree, it is possible to make inferences about the phylogenetic signal associated with the trait (the lower the variance, the better the tree fits the tip data).

D.2 Estimators of Blomberg et al.

A problem remains however with the independent contrasts, which is that the variance in contrasts strongly depends on the size and the shape of the tree. Blomberg et al. [22] derive a solution to this problem by comparing the candidate tree inferred from the data to 1000 'random' trees (which have the same shape as the original tree but in which the tips values are randomly mixed). This allows one to test whether the variance in the contrast values from the candidate tree significantly differs from the variance one would expect from a tree with the same shape. Blomberg et al. then introduce a more precise estimator for phylogenetic signal, K, which corrects for the size and the shape of the tree in order to facilitate comparisons among trees. If $K = 0$, it means that a star phylogeny (where all the tips are independent) would be the best to explain the observed data, i.e. that there is no phylogenetic signal. If $K = 1$, it means that species resemble each other as expected under Brownian motion evolution of the trait along the candidate tree.

Formally, one of the assumptions made with the K statistics, or with the independent contrast in general, is that the trait evolves on the tree under a Brownian motion process (with a heritability of 1). In reality, other evolutionary processes can take place. A way to model these processes is to modify the branch length. Blomberg et al. develop another estimator, d, which describes the branch length transformation under an Ornstein-Uhlenbeck process (which models stabilizing selection) that best reflects the data. If $d = 0$, it means that a star phylogeny best reflects the data (i.e. that there is no phylogenetic signal). If $d = 1$, the candidate tree best reflects the data. The significance of d is also estimated through a randomisation procedure. Blomberg et al. show that K and d lead to similar qualitative (but not necessarily quantitative) estimates of phylogenetic signal.

Each estimator has advantages and disadvantages. K has the advantage of being robust: removing some of the tips of the tree has little effect on its value (for spVL, removing at random, 10 times, 14 of the 134 patients yields an average value of K of 0.57 ± 0.09 instead of 0.59). Moreover, the value of K corrects for the size and the shape of the tree, which makes comparisons between trees easier. The problem is that K tends to underestimate phylogenetic signal if the phylogeny is too large (more than 200 patients). d has the advantage to be based on an evolution model different than Brownian motion. However, this estimator is less stable than K and does not correct for tree size and shape.

K and d were estimated using Matlab programs (Physig.m and Physigou.m) developed by Garland, Ives and Blomberg [22]. The candidate trees were processed through a program written in R and available from SA upon request.

D.3 Estimators of Pagel and Lynch

Pagel [23,24] developed a test for phylogenetic signal based on a generalised least squares approach. The corresponding estimator is denoted λ. The test assumes a constant-variance random effects model of trait evolution. It then scales the variance-covariance matrix describing the phylogeny by a factor λ. The value of λ that leads to the best fit of the tip data is estimated by a maximum likelihood test. If

$\lambda = 0$, we have phylogenetic independence and if $\lambda = 1$ we have phylogenetic dependence expected under a constant-variance model. Pagel's λ was estimated using the software BayesTraits 1.0 [25]. We used the Continuous option, a random walk process and analysed one trait at a time.

For completeness, note that a similar approach developed by Lynch [26,27] and called the phylogenetic mixed model splits the variance of the trait data into two components, one being a heritable component resulting from phylogenetic associations and the other being a residual variation that is independent of phylogeny. Phylogenetic signal in the data is estimated by the ratio of the two components of variance.

λ has the advantage to handle well large phylogenies. The problem is that λ is less robust than K: for instance, removing 10% of the tree tips randomly can lead to the disappearance of the signal (see Text S1). This is why we study the median value of λ on a set of trees (typically the output of a Bayesian computation of the phylogeny). All the estimators we use are described in further details in Text S1 but we encourage the reader to refer to the original papers for a thorough presentation.

E Estimating correlations between traits

We measured correlations between traits by fitting a linear model using generalised least squares model with the 'gls' function of the 'nlme' package in R. The advantage of the gls function is that it can incorporate correlations between data points. This is exactly what we have with our patients and the correlation is given by the phylogeny. We used functions of the 'ape' package of R [16] to estimate the covariance matrix, i.e. the correlation structures, associated with the tree and import it into the gls function. The two functions we use to estimate the correlation structures are corBrownian, which assumes a Brownian motion evolution on the tree [21], and corrPagel, which uses the same variances as corBrownian but multiplies the covariances by Pagel's λ [24]. Note that the parameter used in these functions (e.g. λ) is 1 by default but it can be varied. Lavin et al. [28] for instance use the value of λ obtained after a maximum likelihood test. However, using such values of λ did not affect the results significantly.

References

1. The Swiss HIV Cohort Study (2010) Cohort Profile: The Swiss HIV Cohort Study. Int J Epidemiol in press: doi: 10.1093/ije/dyp321.

2. Ledergerber B, von Overbeck J, Egger M, Luthy R (1994) The Swiss HIV Cohort Study: rationale, organization and selected baseline characteristics. Soz Praventivmed 39: 387–394.

3. Ledergerber B, Egger M, Opravil M, Telenti A, Hirschel B, et al. (1999) Clinical progression and virological failure on highly active antiretroviral therapy in HIV-1 patients: a prospective cohort study. Lancet 353: 863-8.

4. von Wyl V, Yerly S, Böni J, Bürgisser P, Klimkait T, et al. (2007) Emergence of HIV-1 drug resistance in previously untreated patients initiating combination antiretroviral treatment: a comparison of different regimen types. Arch Intern Med 167: 1782-90.

5. Kouyos RD, von Wyl V, Yerly S, Böni J, Taffé P, et al. (2010) Molecular epidemiology reveals long-term changes in HIV type 1 subtype B transmission in Switzerland. J Infect Dis 201: 1488-97.

6. Ariën KK, Vanham G, Arts EJ (2007) Is HIV-1 evolving to a less virulent form in humans? Nat Rev Microbiol 5: 141–151.

7. Wolbers M, Bucher HC, Furrer H, Rickenbach M, Cavassini M, et al. (2008) Delayed diagnosis of HIV infection and late initiation of antiretroviral therapy in the Swiss HIV Cohort Study. HIV Med 9: 397-405.

8. Taffé P, May M, Swiss HIV Cohort Study (2008) A joint back calculation model for the imputation of the date of HIV infection in a prevalent cohort. Stat Med 27: 4835-53.

9. Fraser C, Hollingsworth TD, Chapman R, de Wolf F, Hanage WP (2007) Variation in HIV-1 set-point viral load: epidemiological analysis and an evolutionary hypothesis. Proc Natl Acad Sci USA 104: 17441–17446.

10. Hollingsworth TD, Laeyendecker O, Shirreff G, Donnelly CA, Serwadda D, et al. (2010) HIV-1 transmitting couples have similar viral load set-points in Rakai, Uganda. PLoS Pathog 6: e1000876.

11. Fellay J, Shianna KV, Ge D, Colombo S, Ledergerber B, et al. (2007) A whole-genome association study of major determinants for host control of HIV-1. Science 317: 944–947.

12. Fellay J, Ge D, Shianna KV, Colombo S, Ledergerber B, et al. (2009) Common genetic variation and the control of HIV-1 in humans. PLoS Genet 5: e1000791.

13. Rockstroh JK, Mocroft A, Soriano V, Tural C, Losso MH, et al. (2005) Influence of hepatitis C virus infection on HIV-1 disease progression and response to highly active antiretroviral therapy. J Infect Dis 192: 992-1002.

14. Hirsch MS, Günthard HF, Schapiro JM, Brun-Vézinet F, Clotet B, et al. (2008) Antiretroviral drug resistance testing in adult HIV-1 infection: 2008 recommendations of an International AIDS Society-USA panel. Clin Infect Dis 47: 266-85.

15. Felsenstein J (2004) Inferring phylogenies. Sunderland, MA, USA: Sinauer Associates, Inc.

16. Paradis E, Claude J, Strimmer K (2004) APE: analyses of phylogenetics and evolution in R language. Bioinformatics 20: 289–290.

17. Paradis E (2006) Analysis of phylogenetics and evolution with R. New-York, USA: Springer Verlag.

18. Guindon S, Gascuel O (2003) A simple, fast, and accurate algorithm to estimate large phylogenies by maximum likelihood. Syst Biol 52: 696–704.

19. Drummond AJ, Rambaut A (2007) BEAST: Bayesian evolutionary analysis by sampling trees. BMC Evol Biol 7: 214.

20. Beerenwinkel N, Däumer M, Oette M, Korn K, Hoffmann D, et al. (2003) Geno2pheno: Estimating phenotypic drug resistance from HIV-1 genotypes. Nucleic Acids Res 31: 3850-5.

21. Felsenstein J (1985) Phylogenies and the Comparative Method. Am Nat 125: 1–15.

22. Blomberg SP, Garland TJ, Ives AR (2003) Testing for phylogenetic signal in comparative data: behavioral traits are more labile. Evolution 57: 717–745.

23. Pagel M (1994) Detecting correlated evolution on phylogenies: a general method for the comparative analysis of discrete characters. Proc R Soc Lond B 255: 37–45.

24. Freckleton RP, Harvey PH, Pagel M (2002) Phylogenetic analysis and comparative data: a test and review of evidence. Am Nat 160: 712–726.

25. Barker D, Meade A, Pagel M (2007) Constrained models of evolution lead to improved prediction of functional linkage from correlated gain and loss of genes. Bioinformatics 23: 14–20.

26. Lynch M (1991) Methods for the analysis of comparative data in evolutionary biology. Evolution 45: 1065–1080.

27. Housworth EA, Martins EP, Lynch M (2004) The phylogenetic mixed model. Am Nat 163: 84–96.

28. Lavin SR, Karasov WH, Ives AR, Middleton KM, Garland TJ (2008) Morphometrics of the avian small intestine compared with that of nonflying mammals: a phylogenetic approach. Physiol Biochem Zool 81: 526–550.
